# Discharge after hip fracture surgery in relation to mobilisation timing by patient characteristics: linked secondary analysis of the UK National Hip Fracture Database

**DOI:** 10.1186/s12877-021-02624-w

**Published:** 2021-12-15

**Authors:** Katie J. Sheehan, Aicha Goubar, Finbarr C. Martin, Chris Potter, Gareth D. Jones, Catherine Sackley, Salma Ayis

**Affiliations:** 1 Department of Population Health Sciences, School of Population Health & Environmental Sciences, Faculty of Life Science and Medicine, King’s College London, 2nd Floor Addison House, Guy’s Campus, London, SE1 1UL UK; 2 Guy’s and St. Thomas’s National Health Service Foundation Trust, London, UK

**Keywords:** Fracture neck of femur, Ambulation, Acute care, Key performance indicators, Quality improvement, Audit, Competing risk, Rehabilitation

## Abstract

**Background:**

Early mobilisation leads to a two-fold increase in the adjusted odds of discharge by 30-days compared to late mobilisation. Whether this association varies by patient characteristics identified as reasons for delayed mobilisation is unknown.

**Methods:**

Audit data was linked to hospitalisation records for 133,319 patients 60 years or older surgically treated for hip fracture in England or Wales between 2014 and 2016. Adjusted proportional odds regression models tested whether the cumulative incidences of discharge differed between those mobilised early and those mobilised late for subgroups defined by dementia, delirium, hypotension, prefracture ambulation, and prefracture residence, accounting for the competing risk of death.

**Results:**

Overall, 34,253 patients presented with dementia, 9818 with delirium, and 10,123 with hypotension. Prefracture, 100,983 were ambulant outdoors, 30,834 were ambulant indoors only, 107,144 were admitted from home, and 23,588 from residential care. 1502 had incomplete data for ambulation and 2587 for prefracture residence. 10, 8, 8, 12, and 12% fewer patients with dementia, delirium, hypotension, ambulant indoors only prefracture, or admitted from residential care mobilised early when compared to those who presented without dementia, delirium, hypotension, with outdoor ambulation prefracture, or admitted from home. The adjusted odds ratios of discharge by 30-days postoperatively among those who mobilised early compared with those who mobilised late were 1.71 (95% CI 1.62–1.81) for those with dementia, 2.06 (95% CI 1.98–2.15) without dementia, 1.56 (95% CI 1.41–1.73) with delirium, 2.00 (95% CI 1.93–2.07) without delirium, 1.83 (95% CI, 1.66–2.02) with hypotension, 1.95 (95% CI, 1.89–2.02) without hypotension, 2.00 (95% CI 1.92–2.08) with outdoor ambulation prefracture, 1.80 (95% CI 1.70–1.91) with indoor ambulation only prefracture, 2.30 (95% CI 2.19–2.41) admitted from home, and 1.64 (95% CI 1.51–1.77) admitted from residential care, accounting for the competing risk of death.

**Conclusion:**

Irrespective of dementia, delirium, hypotension, prefracture ambulation or residence, early compared to late mobilisation increased the likelihood of hospital discharge by 30-days postoperatively. However, fewer patients with dementia, delirium, or hypotension, poorer prefracture ambulation, or from residential care mobilised early. There is a need reduce this care gap by ensuring sufficient resource to enable all patients to benefit from early mobilisation.

**Supplementary Information:**

The online version contains supplementary material available at 10.1186/s12877-021-02624-w.

## Background

Hip fracture is associated with poor outcomes including postoperative complications, [1] failure to recover ambulatory ability, [2] discharge to a new more dependent setting, [3] and mortality [4]. Most hip fractures are treated surgically with the goals of reducing pain and re-establishing ambulation [5]. In 2011, the United Kingdom (UK) National Institute for Health and Care Excellence published guidance which suggested patients are offered a physiotherapist assessment the day after surgery and mobilisation (observed ability to sit or stand out of bed, with or without assistance) thenceforth at least once a day unless contraindicated [6]. A UK national audit report in 2016 demonstrated that 21% of patients were not enabled to mobilise within this time [7]. Internationally this figure is considerable higher. Among countries with national audit of hip fracture, up to 45% of patients do not mobilise within the recommended time [8]. It is possible this is even higher for countries where national audit is not in place.

In 2017 the UK Chartered Society of Physiotherapy commissioned a Physiotherapy Hip Fracture Sprint Audit which collected data reporting reasons patients fail to mobilise by the day after surgery from physiotherapists [9]. Reasons included patient specific clinical characteristics: agitation or refusal (potentially due to dementia or delirium), hypotension, and poor prefracture ambulation [9]. These conditions may be more frequently observed among patients admitted from residential care compared to those admitted from home. These possible reasons were reinforced by a public and patient involvement group established to inform the current research. The group members had experienced, or cared for someone who experienced, hip fracture.

A recent analysis indicated early mobilisation (within 36-h of surgery) led to a near two-fold increase in the adjusted odds of discharge by 30-days postoperatively when compared to late mobilisation (beyond 36-h) [10]. The extent to which the aforementioned patient characteristics (agitation or refusal, hypotension, prefracture ambulation, prefracture residence) influence the strength of the association between mobilisation timing and discharge is unknown. Therefore, the purpose of this study is to report on analyses of the associations between mobilisation timing and discharge among groups of patients defined by dementia, delirium, hypotension, prefracture ambulation, and prefracture residence. In this way this study sought to distinguish the contributions of delayed mobilisation from patient characteristics identified as potential barriers to early mobilisation to variations in discharge.

## Methods

### Study design and approvals

This study is reported in adherence to the REporting of studies Conducted using Observational Routinely-collected Data (RECORD) statement [11]. This study received National Health Service (NHS) Health Research Authority and Health and Care Research Wales approval (IRAS Project ID: 230215). The study did not require NHS Research Ethics Committee approval as it involves secondary analysis of pseudonymized data i.e. the authors do not have access to the database population used to create the pseudonymized study population.

### Study setting and population

The UK National Hip Fracture Database (NHFD) assembles data on the characteristics of 95% of patients aged 60 years and older with hip fracture and the care they received following admission to acute hospital in England or Wales (UK) [12] Data from the hospital episode is entered by the clinical team at each hospital and approved by a nominated lead consultant geriatrician prior to submission to the NHFD website. Individual patient NHFD data were linked to hospital episode statistics for England and the patient episode database for Wales for additional data on comorbidities, ethnicity, neighbourhood deprivation and mortality. Further details on data cleaning and person-level linkage across databases are described elsewhere [10]. Data were submitted to the NHFD for 170,970 patients surgically-treated for a non-pathological first hip fracture between January 1, 2014 and December 31, 2016. Of these, patients with some ambulation prefracture (*n* = 168,586) and complete data for the exposure and outcome (*n* = 133,319) were selected for analysis. Differences between patients with and without exposure and outcome data are presented in Supplementary File 1, Table S1.

### Primary outcome

The primary outcome was discharge from acute hospital identified from discharge destination codes of the NHFD: own home/sheltered housing, residential care, nursing home, or long-term care hospital. The time to discharge was estimated as the number of days from surgery to discharge, inhospital death, or 30 days, whichever came first.

### Exposure

The primary exposure was timing of mobilisation (observed ability to sit or stand out of bed, with or without assistance) defined by the NHFD as early (on the day of or day after surgery i.e. within 36-h of surgery) or late (more than 2 days of surgery i.e. after 36-h of surgery) [12]. Once this process is observed it may be repeated by the patient independently, or with support from members of the multidisciplinary team, reducing dependence for ambulation and preparing for discharge.

### Subgroups

Diagnoses of dementia and/or delirium were used as explanatory proxies for the ‘agitation or refusal’ category reported as a potential barrier to early mobilisation in the Physiotherapy Hip Fracture Sprint Audit [9]. International Classification of Disease (ICD)-10 codes were used to identify patients with dementia [ICD-10: E100-E108, E110-E118, E130-E138, E140-E148], delirium [ICD-10: F05], and/or hypotension [ICD-10: I95] during their admission with hip fracture or an admission in the year prior to their hip fracture. Prefracture ambulation was classified as outdoors [NHFD: ambulation without aids, ambulation outdoors with one aid, ambulation outdoors with two aids or frame] or indoors only [NHFD: some indoor ambulation but never goes outside without help]. Prefracture residence was classified as home (NHFD: own home, sheltered housing) or residential care (NHFD: nursing care, residential care).

### Potential confounders

The following were considered potential confounders for our analysis: age, [13] sex, [13] ethnicity (White, Caribbean or African or any mixed Black background, Asian or Asian British or any mixed Asian background, Any other mixed background), [14] fracture type (intracapsular, intertrochanteric/subtrochanteric), [13] deprivation (Index of Multiple Deprivation decile groups), [15] American Society of Anaesthesiologists (ASA) grade, [16] prefracture residence (own home/sheltered housing, nursing care/residential care, other (rehabilitation unit/acute hospital/already in hospital/this hospital site/other hospital site of this trust/other hospital trust)) (not for prefracture residence subgroup or additive analysis), [13] prefracture ambulation (indoor only, outdoor) (not for prefracture ambulation subgroup analysis or additive analysis), [17] timing of surgery (within 36-h target time, not within 36-h target time), [18] procedure type (internal fixation, hemiarthroplasty/arthroplasty), [19] day of admission (Monday-Friday, Saturday-Sunday), [20] and hospital case volume based on the average annual number of surgeries at the admitting hospital (low (quartile of fewest cases), medium (second and third quartile), or high (fourth quartile) volume., [20] and calendar year of admission (2014, 2015, 2016) as a proxy for changes in practice and funding. Adjustments were also made for the following comorbidities: heart failure or pulmonary oedema, [21] chronic obstructive pulmonary disease, [22] ischaemic heart disease (acute or chronic), [23] cardiac dysrhythmias, [24] hypertension, [25] hypotension (not for hypotension subgroup analysis), [26] diabetes with complication, [27] Alzheimer’s or dementia (not for dementia subgroup analysis or additive analysis), [28] depression, [29] and delirium (not for delirium subgroup analysis or additive analysis) [29]. The ICD-10 codes used to identify each comorbidity are available elsewhere [10].

### Statistical analysis

Continuous patient, structure, and process characteristics were described as median and interquartile ranges, and categorical characteristics as counts and proportions, overall and by timing of mobilisation for the entire study cohort and for subgroups defined by dementia, delirium, prefracture ambulation and hypotension. The Wilcoxon Rank Sum test was used to compare distribution of continuous variables and the χ^2^ test to compare proportions by timing of mobilisation overall and for each subgroup. The daily rate of discharge by mobilisation timing for each subgroup was calculated by dividing the number of corresponding events by the total number of inpatient days. The cumulative incidence of discharge was estimated as a function of postoperative day, with inhospital death as a competing event, by timing of mobilisation for each subgroup. Hospital stays ending with loss to follow-up (NHFD discharge destination of rehabilitation unit, acute hospital or unit) and stays greater than 30 postoperative days were right-censored [30]. The Pepe-Mori 2-sample test [31] and proportional odds regression models [32] were used to test whether the cumulative incidences of discharge differed between those mobilised early and those mobilised late, for each subgroup. It is likely subgroups do not occur in isolation, for example, those with dementia may also more likely present from residential care. Therefore, a further analysis was completed to consider the additive role of subgroups which significantly influenced the association between mobilisation timing and discharge in the individual analyses. Results were described by 30-day risk differences [33] and by odds ratios [34].

All analyses were completed in R for statistical computing [35] using the following packages: CIFsmry, [36] cmprsk, [37] prodlim [38] and geepack [39].

Sensitivity analysis.

The potential influence of missing data in the exposure and potential confounders was explored through multiple imputation by chained equations (MICE) using MICE R package and analysis model [40, 41]. We replaced missing values with a random sample of imputed values and estimated the 30-day risk differences and odds ratios in 25 distinct imputed datasets to reduce sampling variability while limiting the loss of power for assessing the timing-discharge association to no more than 1% [40, 42]. We combined results across imputed datasets using Rubin’s rules [43].

## Results

### Study population

Data was analysed for 133,319 patients aged 60 years or older who underwent surgery for nonpathological first hip fracture at an English or Welsh hospital between 2014 and 2016. Most of these patients were women (97,001 [72.8%]), admitted from home (107,144 [80.4%]), and with a median age of 84 years (IQR 77–89). Just over half presented with an ASA grade III (73,694 [55.3%]) and the most common comorbidities were hypertension (64,673 [48.5%]), Alzheimer’s or dementia (34,253 [25.7%]), and cardiac dysrhythmias (26,319 [19.7%]) (Table 1). By day 30 after surgery, 70,253 (53%) stays ended with discharge, 5581 (4%) stays ended with hospital death, 44,115 (33%) had right-censoring events, and 13,370 (10%) stays were longer than 30 days. Overall, 106,722 (79%) patients mobilised early. Characteristics of patients by timing of mobilisation for each subgroup are presented in Supplementary File 1.

**Table 1:** Characteristics of patients surgically treated for non-pathological first hip fracture overall and by timing of mobilisation

		All (***N*** = 133,319) n(%)	early mobilisation (***N*** = 105,651) n(%)	delayed mobilisation (***N*** = 27,668) n(%)
Age (years) median [IQR] ^e^] *		84 [77–89]	84 [77–89]	85 [79–90]
Sex*	Female	97,001 (72.8)	77,299 (79.7)	19,702 (20.3)
	Male	36,316 (27.2)	28,351 (78.1)	7965 (21.9)
	Missing	2 (0.0)	1 (50.0)	1 (50.0)
Ethnicity*	White	94,195 (70.7)	75,116 (79.7)	19,079 (20.3)
	Caribbean or African (Black or Black British) or any mixed black background	221 (0.2)	145 (65.6)	76 (34.4)
	Asian or Asian British or any mixed Asian background	1173 (0.9)	905 (77.2)	268 (22.8)
	Any other Mixed background	24 (0.0)	18 (75.0)	6 (25.0)
	Missing	37,706 (28.3)	29,467 (78.1)	8239 (21.9)
Deprivation*	Least deprived 10%	9874 (7.4)	7735 (78.3)	2139 (21.7)
	Less deprived 10–20%	9742 (7.3)	7546 (77.5)	2196 (22.5)
	Less deprived 20–30%	10,579 (7.9)	8205 (77.6)	2374 (22.4)
	Less deprived 30–40%	11,379 (8.5)	8864 (77.9)	2515 (22.1)
	Less deprived 40–50%	11,954 (9.0)	9416 (78.8)	2538 (21.2)
	More deprived 40–50%	12,616 (9.5)	9940 (78.8)	2676 (21.2)
	More deprived 30–40%	12,400 (9.3)	9821 (79.2)	2579 (20.8)
	More deprived 20–30%	12,035 (9.0)	9610 (79.9)	2425 (20.1)
	More deprived 10–20%	11,929 (8.9)	9591 (80.4)	2338 (19.6)
	Most deprived 10%	11,307 (8.5)	9138 (80.8)	2169 (19.2)
	Missing	19,504 (14.6)	15,785 (80.9)	3719 (19.1)
Prefracture ambulation*	Outdoor ambulation	100,983 (75.7)	82,919 (82.1)	18,064 (17.9)
	Indoor ambulation only	30,834 (23.1)	21,663 (70.3)	9171 (29.7)
	Missing	1502 (1.1)	1069 (71.2)	433 (28.8)
Fracture type*	Intracapsular	78,830 (59.1)	63,022 (79.9)	15,808 (20.1)
	Intertrochanteric	46,566 (34.9)	36,745 (78.9)	9821 (21.1)
	Subtrochanteric	7864 (5.9)	5836 (74.2)	2028 (25.8)
	Missing	59 (0.0)	48 (81.4)	11 (18.6)
Surgery timing*	Within target time of 36 h	95,542 (71.7)	76,489 (80.1)	19,053 (19.9)
	Not within target time	29,498 (22.1)	22,569 (76.5)	6929 (23.5)
	Missing	8279 (6.2)	6593 (79.6)	1686 (20.4)
Procedure type*	Internal fixation	64,845 (48.6)	51,500 (79.4)	13,345 (20.6)
	Hemiarthroplasty	57,539 (43.2)	44,514 (77.4)	13,025 (22.6)
	Total Hip replacement	10,393 (7.8)	9238 (88.9)	1155 (11.1)
	Missing/Other	542 (0.4)	399 (73.6)	143 (26.4)
Calendar year of surgery*	2014	31,205 (23.4)	24,373 (78.1)	6832 (21.9)
	2015	53,448 (40.1)	42,734 (80.0)	10,714 (20.0)
	2016	48,666 (36.5)	38,544 (79.2)	10,122 (20.8)
Weekday of admission*	Weekday	89,840 (67.4)	70,990 (79.0)	18,850 (21.0)
	Weekend	41,357 (31.0)	33,156 (80.2)	8201 (19.8)
	Missing	2122 (1.6)	1505 (70.9)	617 (29.1)
Hospital volume*^c^	High volume	68,323 (51.2)	53,967 (79.0)	14,356 (21.0)
	Medium volume	31,553 (23.7)	25,448 (80.7)	6105 (19.3)
	Low volume	33,443 (25.1)	26,236 (78.4)	7207 (21.6)
ASA grade*^b^	I	3101 (2.3)	2819 (90.9)	282 (9.1)
	II	36,499 (27.4)	31,410 (86.1)	5089 (13.9)
	III	73,694 (55.3)	57,483 (78.0)	16,211 (22.0)
	IV	16,515 (12.4)	11,159 (67.6)	5356 (32.4)
	V	275 (0.2)	162 (58.9)	113 (41.1)
	Missing	3235 (2.4)	2618 (80.9)	617 (19.1)
Comorbidities*^a^	Heart failure or pulmonary oedema	12,753 (9.6)	9056 (71.0)	3697 (29.0)
	Chronic obstructive pulmonary	17,107 (12.8)	12,957 (75.7)	4150 (24.3)
	Ischemic heart (acute)	11,369 (8.5)	8538 (75.1)	2831 (24.9)
	Cardiac dysrhythmias	26,319 (19.7)	19,721 (74.9)	6598 (25.1)
	Ischemic heart (chronic)	19,836 (14.9)	14,933 (75.3)	4903 (24.7)
	Hypertension	64,673 (48.5)	51,252 (79.2)	13,421 (20.8)
	Hypotension	10,123 (7.6)	7298 (72.1)	2825 (27.9)
	Diabetes with complication	1627 (1.2)	1221 (75.0)	406 (25.0)
	Alzheimer’s or dementia	34,253 (25.7)	24,810 (72.4)	9443 (27.6)
	Depression	9490 (7.1)	7318 (77.1)	2172 (22.9)
	Delirium	9818 (7.4)	7072 (72.0)	2746 (28.0)
Admitted from location*	Own home/sheltered housing	107,144 (80.4)	87,365 (81.5)	19,779 (18.5)
	Residential care	23,588 (17.7)	16,523 (70.0)	7065 (30.0)
	Other^d^	2567 (1.9)	1747 (68.1)	820 (31.9)
	Missing	20 (0.0)	16 (80.0)	4 (20.0)

* *p* ≤ 0.001

^a^18,624 without comorbidity data. Comorbidities are identified by the presence of ICD-10 diagnosis codes from the hip fracture care spell, or any admissions in the year prior to the hip fracture care spell.

^b^I – normal healthy individual; II – mild systemic disease that does not limit activity; III – severe systemic disease that limits activity but is not incapacitating; IV-incapacitating systemic disease which is constantly life-threatening; V-moribund -not expected to survive 24 h with or without surgery.

^c^low (less than first quartile), medium (second and third quartile), or high (fourth quartile) volume at admission based on the average annual number of surgeries at the admitting hospital.

^d^Rehabilitation unit/acute hospital/already in hospital/this hospital site/other hospital site of this trust/other hospital trust.

^e^IQR: the first and third quartiles respectively.

### Dementia

**Fig. 1:**
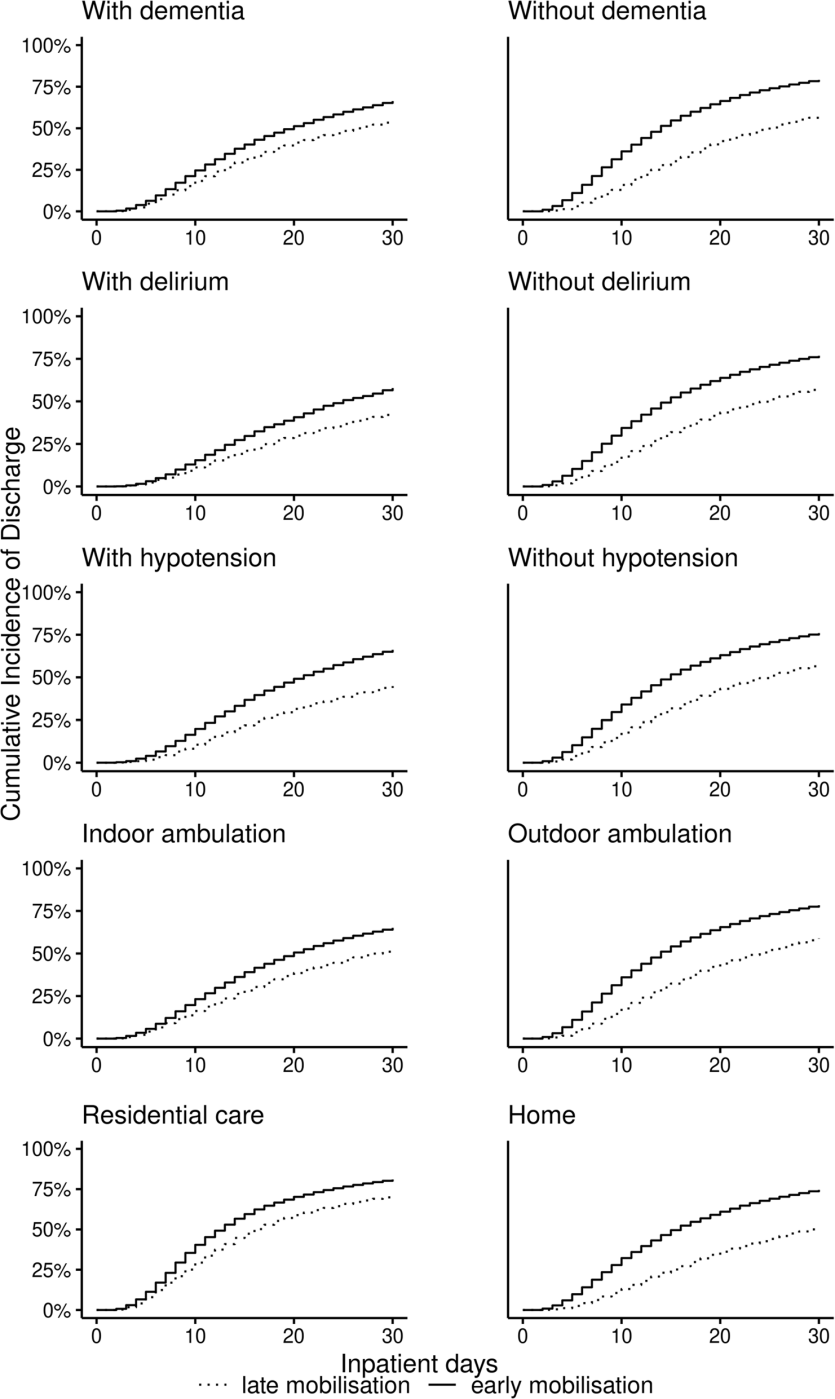
Cumulative incidence of discharge by 30-days postoperatively among patients surgically treated for non-pathological first hip fracture by timing of mobilisation and for subgroups defined by dementia, delirium, hypotension, prefracture ambulation, and prefracture residence

**Fig. 2:**
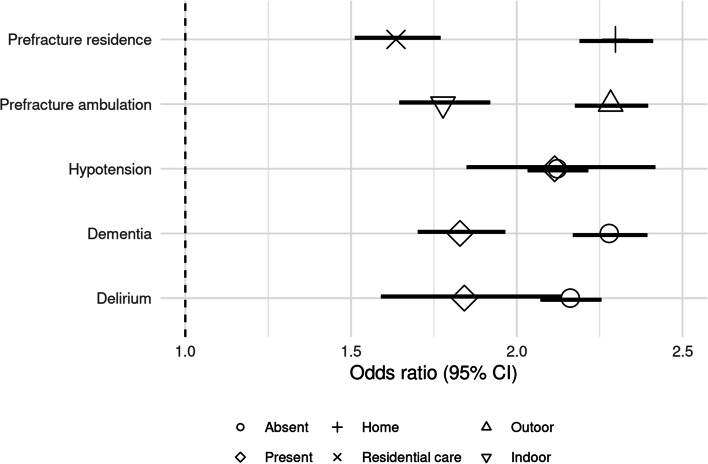
Adjusted odds of discharge by 30-days after surgery for patients mobilised within 36-h compared to those mobilised beyond 36-h of surgery for subgroups defined by dementia, delirium, hypotension, prefracture ambulation, and prefracture residence

In total, 114,695 patients had complete data for the presence or absence of dementia. Of these, 34,253 (30.0%) patients presented with dementia. In total, 65,742 (81.7%) patients without dementia and 24,810 (72.4%) patients with dementia mobilised early. The average rate of discharge per 1000 patient days was 47.0 (95% CI 46.5–47.4) among those mobilised early without dementia, 34.0 (95% CI 33.4–34.6) among those mobilised early with dementia, 26.2 (95% CI 25.5–26.9) among those who mobilised late without dementia, and 26.8 (95% CI 26.0–27.6) among those who mobilised late with dementia (Table 2). There were an additional 217 (95% CI 205–228) and 118 (95% CI 104–132) discharges per 1000 surgeries among patients who mobilised early when compared to those mobilised late, for those without and with dementia respectively (Fig. 1). By 30-days postoperatively, the adjusted odds ratios of discharge among those who mobilised early when compared with those who mobilised late were 2.28 (95% CI 2.17–2.39) for those without dementia and 1.83 (95% CI 1.70–1.97) for those with dementia, accounting for the competing risk of death (Fig. 2, Table 2).

**Table 2:** Discharge by timing of mobilisation among patients surgically treated for non-pathological first hip fracture for subgroups defined by dementia, delirium, hypotension, prefracture ambulation and prefracture residence

Mobilisation timing	No. of patients	No of deaths^a^	No. of live discharges^b^	Live discharge rate (95% CI)^c^	30-day CIF, ^c^ (95% CI)	***p***-value§	Unadjusted OR of CIF (95% CI)	Adjusted OR of CIF (95% CI)^d^
	patients with dementia							
Overall	34,253	17,083	2171	31.9 (31.4–32.4)	630 (624–636)			
Mobilised late	9443	4179	992	26.8 (26–27.6)	545 (534–557)		1.00	1.00
Mobilised early	24,810	12,904	1179	34 (33.4–34.6)	664 (656–671)	< 0.001	1.72 (1.62–1.82)	1.83 (1.70–1.97)
	**patients without dementia**							
Overall	80,442	43,017	2683	42.4 (42.0–42.8)	749 (745–753)			
Mobilised late	14,700	5874	1179	26.2 (25.5–26.9)	574 (564–585)		1.00	1.00
Mobilised early	65,742	37,143	1504	46.9 (46.5–47.4)	791 (787–795)	< 0.001	2.67 (2.57–2.77)	2.28 (2.17–2.39)
	**patients with delirium**							
Overall	9818	4010	775	23.9 (23.2–24.7)	537 (526–549)			
Mobilised late	2746	929	336	19 (17.8–20.3)	436 (414–458)		1.00	1.00
Mobilised early	7072	3081	439	26 (25.1–26.9)	578 (564–592)	< 0.001	1.94 (1.72–2.19)	1.84 (1.59–2.13)
	**patients without delirium**							
Overall	104,877	56,090	4079	40.6 (40.2–40.9)	727 (723–730)			
Mobilised late	21,397	9124	1835	27.5 (27–28.1)	578 (57–587)		1.00	1.00
Mobilised early	83,480	46,966	2244	44.7 (44.2–45.1)	768 (764–772)	< 0.001	2.40 (2.32–2.48)	2.16 (2.07–2.26)
	**patients with hypotension**							
Overall	10,123	4425	759	28.1 (27.3–28.9)	599 (587–611)			
Mobilised late	2825	939	391	19.9 (18.7–21.2)	448 (426–47)		1.00	1.00
Mobilised early	7298	3486	368	31.5 (30.5–32.6)	661 (647–675)	< 0.001	2.39 (2.14–2.66)	2.11 (1.85–2.42)
	**patients without hypotension**							
Overall	104,572	55,675	4095	40.0 (39.6–40.3)	720 (716–723)			
Mobilised late	21,318	9114	1780	27.4 (26.8–27.9)	576 (568–584)		1.00	1.00
Mobilised early	83,254	46,561	2315	43.9 (43.5–44.3)	76 (756–763)	< 0.001	2.36 (2.28–2.44)	2.12 (2.03–2.22)
	**patients with indoor ambulation only prefracture**							
Overall	30,834	14,200	2189	30.2 (29.7–30.7)	611 (604–618)			
Mobilised late	9171	3742	1099	25.1 (24.3–25.9)	523 (511–535)		1.00	1.00
Mobilised early	21,663	10,458	1090	32.6 (32.0–33.2)	651 (643–659)	< 0.001	1.82 (1.72–1.93)	1.78 (1.65–1.92)
	**patients with outdoor ambulation prefracture**							
Overall	100,983	55,344	3265	42.5 (42.1–42.8)	746 (743–750)			
Mobilised late	18,064	7696	1312	27.6 (27–28.2)	588 (579–597)		1.00	1.00
Mobilised early	82,919	47,648	1953	46.5 (46.1–47.0)	784 (780–787)	< 0.001	2.48 (2.4–2.57)	2.28 (2.18–2.4)
	**patients admitted from residential care**							
Overall	23,588	16,252	1428	50.5 (49.7–51.2)	779 (773–784)			
Mobilised late	7065	4448	674	42.7 (41.4–43.9)	707 (696–719)		1.00	1.00
Mobilised early	16,523	11,804	754	54.2 (53.2–55.2)	810 (803–817)	< 0.001	1.68 (1.59–1.79)	1.64 (1.51–1.77)
	**patients admitted from home**							
Overall	107,144	53,284	3938	37.3 (37.0–37.6)	700 (696–703)			
Mobilised late	19,779	6977	1694	22.1 (21.6–22.6)	512 (530–521)		1.00	1.00
Mobilised early	87,365	46,307	2244	41.6 (41.2–42.0)	746 (742–749)	< 0.001	2.85 (2.75–2.96)	2.30 (2.19–2.41)

*Abbreviations: CIF* Cumulative incidence function, *CI* Confidence interval, *OR* Odds ratio

^a^At 30 days from surgery ^b^At 30 days from surgery ^c^Per 1000 patient–days. § Pepe-Mori test *p*-value. Two–sample test compared to mobilised 2 days or more after surgery ^d^Adjusted for age, sex, ethnicity, fracture type, calendar period of admission, timing of surgery, comorbidity (in each subgroup analysis by hypotension, dementia, delirium the corresponding variable is excluded from the adjustment), prefracture residence (except for analysis by prefracture residence subgroup), prefracture ambulation (except for the analysis by prefracture ambulation subgroup), procedure type, day of admission and hospital volume. CIF regression at in-patient days 3, 4, 6, 8, 12, 16, 20, 24, and 30. Results based on a subset of patients with known information for adjustment variables. The analysis of patients with imputation for missing values in adjustment variables is available in supplementary file 4

### Delirium

In total, 114,695 patients had complete data for the presence or absence of delirium. Of these, 9818 (9.4%) patients presented with delirium. In total, 83,480 (79.6%) patients without delirium and 7072 (72.0%) patients with delirium mobilised early. The average rate of discharge per 1000 patient days was 44.7 (95% CI 44.2–45.1) among those mobilised early without delirium, 26.0 (95% CI 25.1–26.9) among those mobilised early with delirium, 27.5 (95% CI 27.0–28.1) among those who mobilised late without delirium, and 19.0 (95% CI 17.8–20.3) among those who mobilised late with delirium (Table 2). There were an additional 190 (95% CI 180–199) and 143 (95% CI 116–169) discharges per 1000 surgeries among patients who mobilised early when compared to those mobilised late, for those without and with delirium respectively (Fig. 1). By 30-days postoperatively, the adjusted odds ratios of discharge among those who mobilised early when compared with those who mobilised late were 2.16 (95% CI 2.07–2.26) for those without delirium and 1.84 (95% CI 1.59–2.13) for those with delirium, accounting for the competing risk of death (Fig. 2, Table 2).

### Hypotension

In total, 114,695 patients had complete data for the presence or absence of hypotension. Of these, 10,123 (8.8%) patients presented with hypotension. In total, 83,254 (79.6%) patients without hypotension and 7298 (72.1%) patients with hypotension mobilised early. The average rate of discharge per 1000 patient days was 43.9 (95% CI 43.5–44.3) among those mobilised early without hypotension, 31.5 (95% CI 30.5–32.6) among those mobilised early with hypotension, 27.4 (95% CI 26.8–27.9) among those who mobilised late without hypotension, and 19.9 (95% CI 18.7–21.2) among those who mobilised late with hypotension (Table 2). There were an additional 213 (95% CI 187–240) and 183 (95% CI 174–193) discharges per 1000 surgeries among patients who mobilised early when compared to those mobilised late, for those without and with hypotension respectively (Fig. 1). By 30-days postoperatively, the adjusted odds ratios of discharge among those who mobilised early when compared with those who mobilised late were 2.12 (95% CI, 2.03–2.22) for those without hypotension and 2.11 (95% CI, 1.85–2.42) for those with hypotension, accounting for the competing risk of death (Fig. 2, Table 2).

### Prefracture ambulation

In total, 131,817 patients had complete data for indoor only or outdoor ambulation prefracture. Of these, 100,983 (76.6%) patients presented with outdoor ambulation prefracture and 30,834 (23.4%) patients presented with indoor ambulation only prefracture. In total, 82,919 (82.1%) patients without outdoor ambulation prefracture and 21,663 (70.3%) patients with indoor ambulation only prefracture mobilised early. The average rate of discharge per 1000 patient days was 46.5 (95% CI 46.1–47.0) among those mobilised early with outdoor ambulation prefracture, 32.6 (95% CI 32.0–33.2) among those mobilised early with indoor ambulation only prefracture, 27.6 (95% CI 27.0–28.2) among those who mobilised late with outdoor ambulation prefracture, and 25.1 (95% CI 24.3–25.9) among those who mobilised late with indoor ambulation only prefracture (Table 2). There were an additional 195 (95% CI 185–205) and 128 (95% CI 113–143) discharges per 1000 surgeries among patients who mobilised early when compared to those mobilised late, for those with outdoor ambulation and indoor ambulation only prefracture respectively (Fig. 1). By 30-days postoperatively, the adjusted odds ratios of discharge among those who mobilised early when compared with those who mobilised late were 2.28 (95% CI 2.18–2.40) for those with outdoor ambulation prefracture and 1.78 (95% CI 1.65–1.92) for those with indoor ambulation only prefracture, accounting for the competing risk of death (Fig. 2, Table 2).

### Prefracture residence

In total, 130,732 patients had complete data for home or residential care prefracture residence. Of these, 107,144 (82.0%) patients were admitted from home and 23,588 (18.0%) patients were admitted from residential care. In total, 87,365 (81.5%) patients admitted from home and 16,523 (70.1%) patients admitted from residential care mobilised early. The average rate of discharge per 1000 patient days was 41.6 (95% CI 41.2–42) among those mobilised early and admitted from home, 54.2 (95% CI 53.2–55.2) among those mobilised early and admitted from residential care, 22.1 (95% CI 21.6–22.6) among those who mobilised late and admitted from home, and 42.7 (95% CI 41.4–43.9) among those who mobilised late and admitted from residential care (Table 2). There were an additional 234 (95% CI 224–244) and 103 (95% CI 89–117) discharges per 1000 surgeries among patients who mobilised early when compared to those mobilised late, among those admitted from home and those admitted from residential care respectively (Fig. 1). By 30-days postoperatively, the adjusted odds ratios of discharge among those who mobilised early when compared with those who mobilised late were 2.30 (95% CI 2.19–2.41) among those admitted from home and 1.64 (95% CI 1.51–1.77) among those admitted from residential care, accounting for the competing risk of death (Fig. 2, Table 2).

### Dementia, delirium, prefracture ambulation, and prefracture residence

**Fig. 3:**
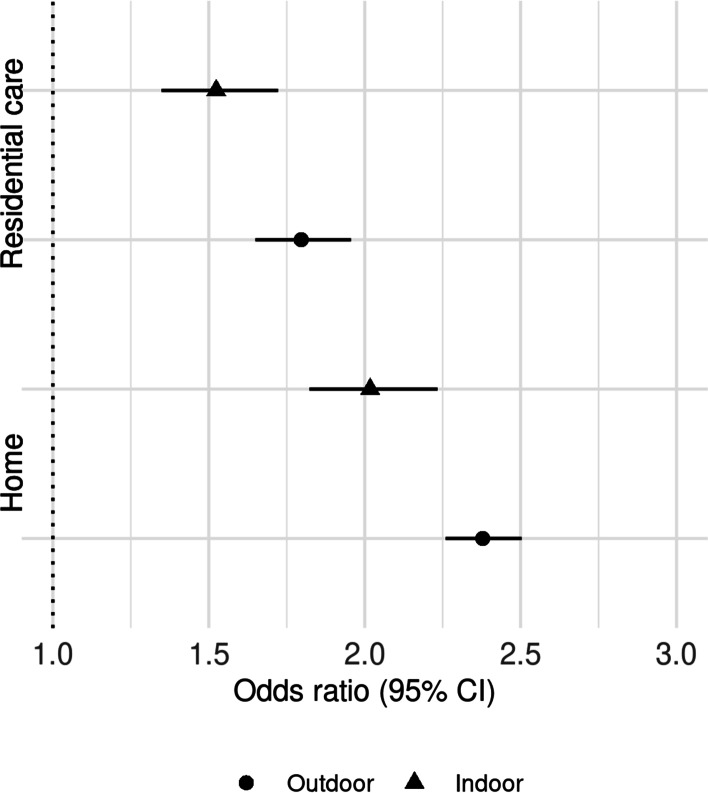
Discharge by timing of mobilisation among patients surgically treated for non-pathological first hip fracture from additive model including mobilisation timing by prefracture mobility and residence

In total, 118,315 patients had complete data for all of the following: the presence or absence of dementia and/or delirium, indoor only or outdoor ambulation prefracture, and home or residential care prefracture residence. Odds ratios for all combinations of these variables are available in Supplementary File 3. From these models, in the presence of prefracture ambulation and residence, delirium and dementia did not alter association between early mobilisation and discharge (Supplementary File 3, S3–1, S3–2). Accounting for the competing risk of death, the adjusted odds ratios of discharge among those who mobilised early when compared to those who mobilised late were 2.38 (95% CI 2.26–2.50) for those with outdoor ambulation prefracture admitted from home, 2.02 (95% CI 1.82–2.23) for those with indoor ambulation only prefracture admitted from home, 1.80 (95% CI 1.65–1.96) for those with outdoor ambulation prefracture admitted from residential care, and 1.52 (95% CI 1.35–1.72) for those with indoor ambulation only prefracture admitted from residential care (Fig. 3, Table 3).

**Table 3:** Discharge by timing of mobilisation among patients surgically treated for non-pathological first hip fracture from additive model including prefracture ambulation and residence

Residence prefracture	Ambulation prefracture	Mobilisation	No. of patients	No of deaths^a^	No. of live discharges ^b^	Unadjusted OR of CIF (95% CI)	Adjusted OR of CIF (95% CI)^c^
	Indoor only	Delayed	3925	412	2483	1	1
		Early	8054	400	5767	1.46 (1.33–1.61)	1.52 (1.35–1.72)
Residential care	Outdoor	Delayed	2974	241	1861	1	1
		Early	8148	340	5818	1.81 (1.7–1.94)	1.8 (1.65–1.96)
Home	Indoor only	Delayed	4911	639	1200	1	1
		Early	13,039	653	4540	2.27 (2.1–2.45)	2.02 (1.82–2.23)
	Outdoor	Delayed	14,640	1015	5719	1	1
		Early	73,637	1548	41,464	2.81 (2.71–2.92)	2.38 (2.26–2.5)

*Abbreviations: CIF* Cumulative incidence function, *CI* Confidence interval, *OR* Odds ratio

^a^At 30 days from surgery

^b^At 30 days from surgery

^c^Adjusted for age, sex, ethnicity, fracture type, calendar period of admission, timing of surgery, comorbidity, procedure type, day of admission and hospital volume. CIF regression at in-patient days 3, 4, 6, 8, 12, 16, 20, 24, and 30. Results based on a subset of patients with known information for adjustment variables. The analysis of patients with imputation for missing values in adjustment variables in available in supplementary file 4

§Pepe-Mori test *p*-value. Two–sample test compared to mobilised 2 days or more after surgery

### Sensitivity analysis

Full detail of results of imputation for missing exposure, potential confounder, and subgroup data are presented in Supplementary File 4. Results of these analyses yielded similar estimates to those of the complete case analysis.

## Discussion

### Main findings

Irrespective of dementia, delirium, hypotension, prefracture ambulation or residence, early mobilisation increased the adjusted odds of hospital discharge by 30-days postoperatively compared to late mobilisation, accounting for the competing risk of inhospital death. The increased rate of discharge was greatest for those without dementia or delirium, able to walk outdoors and admitted from home. The association between mobilisation timing and discharge was similar for those presenting with and without hypotension. Additive modeling suggested only prefracture ambulation and residence (considered together) influenced the association between mobilisation timing and discharge. This modeling suggested patients admitted from home with better ambulation were more likely to be discharged early following early mobilisation than those admitted from residential care with poorer ambulation.

### Comparison with other literature

The current study demonstrated a beneficial association between early mobilisation and discharge for all patient subgroups considered individually and together. However, 10, 8, 8, 12, and 12% fewer patients with dementia, delirium, hypotension, with indoor ambulation only prefracture, or admitted from residential care mobilised early when compared to those who presented without dementia, delirium, hypotension, with outdoor ambulation prefracture, or admitted from home. There is a need to determine underlying mechanisms for the care access gap noted within subgroups in the current study and to address any potential inequities in provision should they become apparent.

Consistency in the access and delivery of physical activity interventions (including mobilising) has been observed in a UK cohort study of hospitalised older people irrespective of their frailty or cognitive status [44]. Despite this consistency, outcomes were poorer in patients with cognitive impairment suggestive of a need to target not only what is offered to patients, but how [44]. Indeed, a recent systematic review reported a positive association between rehabilitation and functional outcomes after hip fracture surgery among patients with cognitive impairment when the approach was tailored to the differing needs of these patients [45]. A tailored approach for patients with dementia or delirium may require additional resources (e.g. staffing numbers, staff expertise, and/or equipment) for safe and effective mobilisation from bed postoperatively compared to those without these conditions. These resources may not be consistently available (e.g. on weekends) and contribute to delays [46].

Alternatively, patients with dementia, delirium, or hypotension, with poorer ambulation, or from residential care may be underprioritized for early mobilisation due to a perceived lack of potential. For example, physiotherapists reported patients with dementia are often prejudged as having limited ‘potential’ after hip fracture leading to failures to attempt to engage these patients in rehabilitation [47]. This is despite finding from the current study which suggest a benefit of early mobilisation on time to discharge after hip fracture surgery. A judgement of limited potential may prevent access to rehabilitation further along the care pathway where patients with dementia have 4.3 times lower odds of transfer to hospital based rehabilitation following hip fracture compared to those without dementia [48]. Further, in the UK only 70% of hospitals have access to physiotherapy follow-up in residential care where therapy input is already limited across residents [49].

The finding that those who presented without dementia or delirium, with better ambulation, or from home gained most from early mobilisation is consistent with previous research where a greater risk of inhospital [50] and 6-month mortality [51] following delayed mobilisation was observed for patients presenting with poorer prefracture function compared to those with better prefracture function. Kenyon-Smith et al. reported early mobilisation reduced the rate of postoperative complications only for those with poor premorbid health (composite measure of age, mobility and comorbidity count) [52]. This suggests the underlying mechanism for the reported associations between mobilisation timing and discharge/death may vary depending on patient characteristics. Irrespective of the differential associations reported across patient subgroups, there is compelling evidence for increased discharge and reduced mortality among all patients when mobilised early compared to those mobilised late [50, 51].

### Limitations

The exposure was a binary indicator of timing of mobilisation – early or late. A continuous measure was not available nor was data related to subsequent mobilisation during the hospital stay. These data may provide further insight to the association between mobilisation timing across subgroups. The analysis was adjusted for known confounders where data was available. However, there was the potential for unmeasured confounding where data was not available. For example, discharge may be influenced by the presence of other conditions such as stroke, other process of care during the acute stay such as weight bearing status in relation specifically to the fracture stabilisation, [53] and/or the occurrence of inhospital postoperative complications [54]. Dementia, delirium and hypotension subgroups were classified according to the presence or absence of ICD-10 diagnosis codes in hospitalisation records. These codes may be subject to underreporting [55]. This may have led to an underestimation of the timing-discharge association within subgroups due to random misclassification whereby the underreporting of conditions was likely similar across early and delayed mobilisation groups. Further, it was not possible to determine disease stage/symptom severity from ICD-10 diagnosis codes which may influence the association between timing and discharge across subgroups. ICD-codes were identified from hospitalisation records during the hip fracture admission or in the year prior to admission. This may have led to an overestimation of the proportion of patients presenting with delirium and/or hypotension during the hip fracture episode, and an underestimation of the potential effect of these conditions on the timing-outcome association. There was potential for bias due to exclusion of patients with missing data for the exposure, potential confounders, subgroups, and/or outcomes. This was addressed with sensitivity analyses whereby missing data for the exposure, potential confounders and subgroups were imputed. The analyses yielded similar results between complete case analysis and imputed analyses and we are therefore confident that exclusion bias was negligible.

## Conclusion

Mobilisation within 36-h of surgery increased the odds of discharge by 30-days for patients irrespective of dementia, delirium, hypotension, prefracture ambulation or residence. Despite this, fewer patients presenting with dementia, delirium, with poorer prefracture ambulation, or from residential care mobilised early when compared to those who did not present with these conditions, had better prefracture ambulation, or were from home. There is a need reduce this care gap by ensuring sufficient resource and appropriate treatment techniques to enable all patients to benefit from early mobilisation.

## Supplementary Information

**Additional file 1.**

## Data Availability

The data that support the findings of this study are available from NHS Digital, NHS Wales Informatics Service, the Royal College of Physician’s Falls and Fragility Fracture Audit programme and Healthcare Quality Improvement Partnership but restrictions apply to the availability of these data, which were used under license for the current study, and so are not publicly available.

## Abbreviations

UK: United Kingdom
RECORD: REporting of studies Conducted using Observational Routinely-collected Data
NHS: National Health Service
NHFD: National Hip Fracture Database
ICD: International Classification of Disease
ASA: American Society of Anaesthesiologists
MICE: Multiple imputation by chained equations
