# Supplementary material for: Discharge after hip fracture surgery in relation to mobilisation timing by patient characteristics: linked secondary analysis of the UK National Hip Fracture Database
Source: BMC Geriatr. 2021 Dec 15;21:694. doi: 10.1186/s12877-021-02624-w (PMC8672496; doi:10.1186/s12877-021-02624-w)
Supplement: Supplementary file 1 — Additional file 1. [file 12877_2021_2624_MOESM1_ESM.docx]

# Supplementary File 1

Table S1-1: Differences between patients with and patients without complete data for exposure and outcome.

|  |  | **All**  **(N=160,668)** | **Complete data**  **(N=133,319)** | **Excluded**  **(N=27,349)** |
| --- | --- | --- | --- | --- |
|  |  | **N(%)** | **N(%)** | **N(%)** |
| Age (years)- median  [Q1-Q3¶] |  | 84 [77-89] | 84 [77-89] | 84 [77-89] |
| Sex | Female | 117303 ( 73.0 ) | 97001 ( 82.7 ) | 20302 ( 17.3 ) |
|  | Male | 43363 ( 27.0 ) | 36316 ( 83.7 ) | 7047 ( 16.3 ) |
|  | Missing | 2 ( 0.0 ) | 2 ( 100.0 ) | 0 ( 0.0 ) |
| Ethnicity | White | 103726 ( 64.6 ) | 94195 ( 90.8 ) | 9531 ( 9.2 ) |
|  | Caribbean or African (Black or Black British) or any mixed black background | 241 ( 0.1 ) | 221 ( 91.7 ) | 20 ( 8.3 ) |
|  | Asian or Asian British or any mixed Asian background | 1300 ( 0.8 ) | 1173 ( 90.2 ) | 127 ( 9.8 ) |
|  | Any other Mixed background | 28 ( 0.0 ) | 24 ( 85.7 ) | 4 ( 14.3 ) |
|  | Missing | 55373 ( 34.5 ) | 37706 ( 68.1 ) | 17667 ( 31.9 ) |
| Deprivation | least deprived 10% | 10921 ( 6.8 ) | 9874 ( 90.4 ) | 1047 ( 9.6 ) |
|  | less deprived 10-20% | 10770 ( 6.7 ) | 9742 ( 90.5 ) | 1028 ( 9.5 ) |
|  | less deprived 20-30% | 11695 ( 7.3 ) | 10579 ( 90.5 ) | 1116 ( 9.5 ) |
|  | less deprived 30-40% | 12473 ( 7.8 ) | 11379 ( 91.2 ) | 1094 ( 8.8 ) |
|  | less deprived 40-50% | 13110 ( 8.2 ) | 11954 ( 91.2 ) | 1156 ( 8.8 ) |
|  | more deprived 40-50% | 13902 ( 8.7 ) | 12616 ( 90.7 ) | 1286 ( 9.3 ) |
|  | more deprived 30-40% | 13700 ( 8.5 ) | 12400 ( 90.5 ) | 1300 ( 9.5 ) |
|  | more deprived 20-30% | 13271 ( 8.3 ) | 12035 ( 90.7 ) | 1236 ( 9.3 ) |
|  | more deprived 10-20% | 13117 ( 8.2 ) | 11929 ( 90.9 ) | 1188 ( 9.1 ) |
|  | most deprived 10% | 12424 ( 7.7 ) | 11307 ( 91.0 ) | 1117 ( 9.0 ) |
|  | Missing | 35285 ( 22.0 ) | 19504 ( 55.3 ) | 15781 ( 44.7 ) |
| Prefracture ambulation | Outdoor ambulation | 116752 ( 72.7 ) | 100983 ( 86.5 ) | 15769 ( 13.5 ) |
|  | indoor ambulation only | 40398 ( 25.1 ) | 30834 ( 76.3 ) | 9564 ( 23.7 ) |
|  | Missing | 3518 ( 2.2 ) | 1502 ( 42.7 ) | 2016 ( 57.3 ) |
| Hip fracture type | Intracapsular | 94908 ( 59.1 ) | 78830 ( 83.1 ) | 16078 ( 16.9 ) |
|  | Intertrochanteric | 55980 ( 34.8 ) | 46566 ( 83.2 ) | 9414 ( 16.8 ) |
|  | Subtrochanteric | 9457 ( 5.9 ) | 7864 ( 83.2 ) | 1593 ( 16.8 ) |
|  | Missing | 323 ( 0.2 ) | 59 ( 18.3 ) | 264 ( 81.7 ) |
| Indicator of surgery within target time of 36 hours | Within target time | 103172 ( 64.2 ) | 95542 ( 92.6 ) | 7630 ( 7.4 ) |
|  | Not within target time | 35511 ( 22.1 ) | 29498 ( 83.1 ) | 6013 ( 16.9 ) |
|  | Missing | 21985 ( 13.7 ) | 8279 ( 37.7 ) | 13706 ( 62.3 ) |
| Procedure type | Internal fixation | 78278 ( 48.7 ) | 64845 ( 82.8 ) | 13433 ( 17.2 ) |
|  | Hemiarthroplasty | 69448 ( 43.2 ) | 57539 ( 82.9 ) | 11909 ( 17.1 ) |
|  | Total Hip replacement | 12260 ( 7.6 ) | 10393 ( 84.8 ) | 1867 ( 15.2 ) |
|  | Missing/Other | 682 ( 0.4 ) | 542 ( 79.5 ) | 140 ( 20.5 ) |
| Calendar year of surgery | 2014 | 42031 ( 26.2 ) | 31205 ( 74.2 ) | 10826 ( 25.8 ) |
|  | 2015 | 69439 ( 43.2 ) | 53448 ( 77.0 ) | 15991 ( 23.0 ) |
|  | 2016 | 49198 ( 30.6 ) | 48666 ( 98.9 ) | 532 ( 1.1 ) |
| Weekday of admission | Weekday (Monday-Friday) | 107478 ( 66.9 ) | 89840 ( 83.6 ) | 17638 ( 16.4 ) |
|  | Weekend (Saturday-Sunday) | 50376 ( 31.4 ) | 41357 ( 82.1 ) | 9019 ( 17.9 ) |
|  | Missing | 2814 ( 1.8 ) | 2122 ( 75.4 ) | 692 ( 24.6 ) |
| Hospital volume§ | High volume | 80709 ( 50.2 ) | 68323 ( 84.7 ) | 12386 ( 15.3 ) |
|  | Medium volume | 40178 ( 25.0 ) | 31553 ( 78.5 ) | 8625 ( 21.5 ) |
|  | Low volume | 39781 ( 24.8 ) | 33443 ( 84.1 ) | 6338 ( 15.9 ) |
| Mobilisation after surgery within target time of day of/day after | Within target time | 105651 ( 65.8 ) | 105651 ( 100.0 ) | 0 ( 0.0 ) |
|  | After target time | 27668 ( 17.2 ) | 27668 ( 100.0 ) | 0 ( 0.0 ) |
|  | Missing | 27349 ( 17.0 ) | 0 ( 0.0 ) | 27349 ( 100.0 ) |
| Mobilisation timing | Within target time | 133319 ( 83.0 ) | 133319 ( 100.0 ) | 0 ( 0.0 ) |
|  | After target time | 27349 ( 17.0 ) | 0 ( 0.0 ) | 27349 ( 100.0 ) |
| ASA grade‡ | I | 3817 ( 2.4 ) | 3101 ( 81.2 ) | 716 ( 18.8 ) |
|  | II | 45052 ( 28.0 ) | 36499 ( 81.0 ) | 8553 ( 19.0 ) |
|  | III | 88012 ( 54.8 ) | 73694 ( 83.7 ) | 14318 ( 16.3 ) |
|  | IV | 19105 ( 11.9 ) | 16515 ( 86.4 ) | 2590 ( 13.6 ) |
|  | V | 325 ( 0.2 ) | 275 ( 84.6 ) | 50 ( 15.4 ) |
|  | Missing | 4357 ( 2.7 ) | 3235 ( 74.2 ) | 1122 ( 25.8 ) |
| Comorbidities† |  |  |  |  |
| heart failure or pulmonary edema | No | 112298 ( 69.9 ) | 101942 ( 90.8 ) | 10356 ( 9.2 ) |
|  | Yes | 14020 ( 8.7 ) | 12753 ( 91.0 ) | 1267 ( 9.0 ) |
|  | Missing | 34350 ( 21.4 ) | 18624 ( 54.2 ) | 15726 ( 45.8 ) |
| chronic obstructive pulmonary diseases | No | 107623 ( 67.0 ) | 97588 ( 90.7 ) | 10035 ( 9.3 ) |
|  | Yes | 18695 ( 11.6 ) | 17107 ( 91.5 ) | 1588 ( 8.5 ) |
|  | Missing | 34350 ( 21.4 ) | 18624 ( 54.2 ) | 15726 ( 45.8 ) |
| ischemic heart disease (acute) | No | 113765 ( 70.8 ) | 103326 ( 90.8 ) | 10439 ( 9.2 ) |
|  | Yes | 12553 ( 7.8 ) | 11369 ( 90.6 ) | 1184 ( 9.4 ) |
|  | Missing | 34350 ( 21.4 ) | 18624 ( 54.2 ) | 15726 ( 45.8 ) |
| cardiac dysrhythmias | No | 97357 ( 60.6 ) | 88376 ( 90.8 ) | 8981 ( 9.2 ) |
|  | Yes | 28961 ( 18.0 ) | 26319 ( 90.9 ) | 2642 ( 9.1 ) |
|  | Missing | 34350 ( 21.4 ) | 18624 ( 54.2 ) | 15726 ( 45.8 ) |
| ischemic heart disease (chronic) | No | 104576 ( 65.1 ) | 94859 ( 90.7 ) | 9717 ( 9.3 ) |
|  | Yes | 21742 ( 13.5 ) | 19836 ( 91.2 ) | 1906 ( 8.8 ) |
|  | Missing | 34350 ( 21.4 ) | 18624 ( 54.2 ) | 15726 ( 45.8 ) |
| hypertension | No | 55208 ( 34.4 ) | 50022 ( 90.6 ) | 5186 ( 9.4 ) |
|  | Yes | 71110 ( 44.3 ) | 64673 ( 90.9 ) | 6437 ( 9.1 ) |
|  | Missing | 34350 ( 21.4 ) | 18624 ( 54.2 ) | 15726 ( 45.8 ) |
| hypotension | No | 115220 ( 71.7 ) | 104572 ( 90.8 ) | 10648 ( 9.2 ) |
|  | Yes | 11098 ( 6.9 ) | 10123 ( 91.2 ) | 975 ( 8.8 ) |
|  | Missing | 34350 ( 21.4 ) | 18624 ( 54.2 ) | 15726 ( 45.8 ) |
| diabetes with complication | No | 124535 ( 77.5 ) | 113068 ( 90.8 ) | 11467 ( 9.2 ) |
|  | Yes | 1783 ( 1.1 ) | 1627 ( 91.3 ) | 156 ( 8.7 ) |
|  | Missing | 34350 ( 21.4 ) | 18624 ( 54.2 ) | 15726 ( 45.8 ) |
| Alzheimer’s or dementia | No | 88637 ( 55.2 ) | 80442 ( 90.8 ) | 8195 ( 9.2 ) |
|  | Yes | 37681 ( 23.5 ) | 34253 ( 90.9 ) | 3428 ( 9.1 ) |
|  | Missing | 34350 ( 21.4 ) | 18624 ( 54.2 ) | 15726 ( 45.8 ) |
| depression | No | 115906 ( 72.1 ) | 105205 ( 90.8 ) | 10701 ( 9.2 ) |
|  | Yes | 10412 ( 6.5 ) | 9490 ( 91.1 ) | 922 ( 8.9 ) |
|  | Missing | 34350 ( 21.4 ) | 18624 ( 54.2 ) | 15726 ( 45.8 ) |
| delirium | No | 115697 ( 72.0 ) | 104877 ( 90.6 ) | 10820 ( 9.4 ) |
|  | Yes | 10621 ( 6.6 ) | 9818 ( 92.4 ) | 803 ( 7.6 ) |
|  | Missing | 34350 ( 21.4 ) | 18624 ( 54.2 ) | 15726 ( 45.8 ) |
| Admitted from location | Own home/sheltered housing | 128941 ( 80.3 ) | 107144 ( 83.1 ) | 21797 ( 16.9 ) |
|  | Nursing care/residential care | 28207 ( 17.6 ) | 23588 ( 83.6 ) | 4619 ( 16.4 ) |
|  | Otherǁ | 3338 ( 2.1 ) | 2567 ( 76.9 ) | 771 ( 23.1 ) |
|  | Missing | 182 ( 0.1 ) | 20 ( 11.0 ) | 162 ( 89.0 ) |

Table S1-2. Differences between patients with known and with missing values of mobilisation timing among patients with known outcome

|  |  | **All**  **(N=160,668)** | **not missing mobilisation**  **(N=133,319)** | **missing mobilisation**  **(N=27,349)** |
| --- | --- | --- | --- | --- |
|  |  | **N(%)** | **N(%)** | **N(%)** |
| Age (years)- median  [Q1-Q3¶] |  | 84 [77-89] | 84 [77-89] | 84 [77-89] |
| Sex | Female | 117303 ( 73.0 ) | 97001 ( 72.8 ) | 20302 ( 74.2 ) |
|  | Male | 43363 ( 27.0 ) | 36316 ( 27.2 ) | 7047 ( 25.8 ) |
|  | Missing | 2 ( 0.0 ) | 2 ( 0.0 ) | 0 ( 0.0 ) |
| Ethnicity | White | 103726 ( 64.6 ) | 94195 ( 70.7 ) | 9531 ( 34.8 ) |
|  | Caribbean or African (Black or Black British) or any mixed black background | 241 ( 0.1 ) | 221 ( 0.2 ) | 20 ( 0.1 ) |
|  | Asian or Asian British or any mixed Asian background | 1300 ( 0.8 ) | 1173 ( 0.9 ) | 127 ( 0.5 ) |
|  | Any other Mixed background | 28 ( 0.0 ) | 24 ( 0.0 ) | 4 ( 0.0 ) |
|  | Missing | 55373 ( 34.5 ) | 37706 ( 28.3 ) | 17667 ( 64.6 ) |
| Deprivation | least deprived 10% | 10921 ( 6.8 ) | 9874 ( 7.4 ) | 1047 ( 3.8 ) |
|  | less deprived 10-20% | 10770 ( 6.7 ) | 9742 ( 7.3 ) | 1028 ( 3.8 ) |
|  | less deprived 20-30% | 11695 ( 7.3 ) | 10579 ( 7.9 ) | 1116 ( 4.1 ) |
|  | less deprived 30-40% | 12473 ( 7.8 ) | 11379 ( 8.5 ) | 1094 ( 4.0 ) |
|  | less deprived 40-50% | 13110 ( 8.2 ) | 11954 ( 9.0 ) | 1156 ( 4.2 ) |
|  | more deprived 40-50% | 13902 ( 8.7 ) | 12616 ( 9.5 ) | 1286 ( 4.7 ) |
|  | more deprived 30-40% | 13700 ( 8.5 ) | 12400 ( 9.3 ) | 1300 ( 4.8 ) |
|  | more deprived 20-30% | 13271 ( 8.3 ) | 12035 ( 9.0 ) | 1236 ( 4.5 ) |
|  | more deprived 10-20% | 13117 ( 8.2 ) | 11929 ( 8.9 ) | 1188 ( 4.3 ) |
|  | most deprived 10% | 12424 ( 7.7 ) | 11307 ( 8.5 ) | 1117 ( 4.1 ) |
|  | Missing | 35285 ( 22.0 ) | 19504 ( 14.6 ) | 15781 ( 57.7 ) |
| Prefracture ambulation | Outdoor ambulation | 116752 ( 72.7 ) | 100983 ( 75.7 ) | 15769 ( 57.7 ) |
|  | indoor ambulation only | 40398 ( 25.1 ) | 30834 ( 23.1 ) | 9564 ( 35.0 ) |
|  | Missing | 3518 ( 2.2 ) | 1502 ( 1.1 ) | 2016 ( 7.4 ) |
| Hip fracture type | Intracapsular | 94908 ( 59.1 ) | 78830 ( 59.1 ) | 16078 ( 58.8 ) |
|  | Intertrochanteric | 55980 ( 34.8 ) | 46566 ( 34.9 ) | 9414 ( 34.4 ) |
|  | Subtrochanteric | 9457 ( 5.9 ) | 7864 ( 5.9 ) | 1593 ( 5.8 ) |
|  | Missing | 323 ( 0.2 ) | 59 ( 0.0 ) | 264 ( 1.0 ) |
| Surgery timing | Within target time | 103172 ( 64.2 ) | 95542 ( 71.7 ) | 7630 ( 27.9 ) |
|  | Not within target time | 35511 ( 22.1 ) | 29498 ( 22.1 ) | 6013 ( 22.0 ) |
|  | Missing | 21985 ( 13.7 ) | 8279 ( 6.2 ) | 13706 ( 50.1 ) |
| Procedure type | Internal fixation | 78278 ( 48.7 ) | 64845 ( 48.6 ) | 13433 ( 49.1 ) |
|  | Hemiarthroplasty | 69448 ( 43.2 ) | 57539 ( 43.2 ) | 11909 ( 43.5 ) |
|  | Total Hip replacement | 12260 ( 7.6 ) | 10393 ( 7.8 ) | 1867 ( 6.8 ) |
|  | Missing/Other | 682 ( 0.4 ) | 542 ( 0.4 ) | 140 ( 0.5 ) |
| Calendar year of surgery | 2014 | 42031 ( 26.2 ) | 31205 ( 23.4 ) | 10826 ( 39.6 ) |
|  | 2015 | 69439 ( 43.2 ) | 53448 ( 40.1 ) | 15991 ( 58.5 ) |
|  | 2016 | 49198 ( 30.6 ) | 48666 ( 36.5 ) | 532 ( 1.9 ) |
| Weekday of admission | Weekday (Monday-Friday) | 107478 ( 66.9 ) | 89840 ( 67.4 ) | 17638 ( 64.5 ) |
|  | Weekend (Saturday-Sunday) | 50376 ( 31.4 ) | 41357 ( 31.0 ) | 9019 ( 33.0 ) |
|  | Missing | 2814 ( 1.8 ) | 2122 ( 1.6 ) | 692 ( 2.5 ) |
| Hospital volume§ | High volume | 80709 ( 50.2 ) | 68323 ( 51.2 ) | 12386 ( 45.3 ) |
|  | Medium volume | 40178 ( 25.0 ) | 31553 ( 23.7 ) | 8625 ( 31.5 ) |
|  | Low volume | 39781 ( 24.8 ) | 33443 ( 25.1 ) | 6338 ( 23.2 ) |
| Mobilisation after surgery within target time of day of/day after | Within target time | 105651 ( 65.8 ) | 105651 ( 79.2 ) | 0 ( 0.0 ) |
|  | After target time | 27668 ( 17.2 ) | 27668 ( 20.8 ) | 0 ( 0.0 ) |
|  | Missing | 27349 ( 17.0 ) | 0 ( 0.0 ) | 27349 ( 100.0 ) |
| ASA grade‡ | I | 3817 ( 2.4 ) | 3101 ( 2.3 ) | 716 ( 2.6 ) |
|  | II | 45052 ( 28.0 ) | 36499 ( 27.4 ) | 8553 ( 31.3 ) |
|  | III | 88012 ( 54.8 ) | 73694 ( 55.3 ) | 14318 ( 52.4 ) |
|  | IV | 19105 ( 11.9 ) | 16515 ( 12.4 ) | 2590 ( 9.5 ) |
|  | V | 325 ( 0.2 ) | 275 ( 0.2 ) | 50 ( 0.2 ) |
|  | Missing | 4357 ( 2.7 ) | 3235 ( 2.4 ) | 1122 ( 4.1 ) |
| Comorbidities |  |  |  |  |
| heart failure or pulmonary edema | No | 112298 ( 69.9 ) | 101942 ( 76.5 ) | 10356 ( 37.9 ) |
|  | Yes | 14020 ( 8.7 ) | 12753 ( 9.6 ) | 1267 ( 4.6 ) |
|  | Missing | 34350 ( 21.4 ) | 18624 ( 14.0 ) | 15726 ( 57.5 ) |
| chronic obstructive pulmonary diseases | No | 107623 ( 67.0 ) | 97588 ( 73.2 ) | 10035 ( 36.7 ) |
|  | Yes | 18695 ( 11.6 ) | 17107 ( 12.8 ) | 1588 ( 5.8 ) |
|  | Missing | 34350 ( 21.4 ) | 18624 ( 14.0 ) | 15726 ( 57.5 ) |
| ischemic heart disease (acute) | No | 113765 ( 70.8 ) | 103326 ( 77.5 ) | 10439 ( 38.2 ) |
|  | Yes | 12553 ( 7.8 ) | 11369 ( 8.5 ) | 1184 ( 4.3 ) |
|  | Missing | 34350 ( 21.4 ) | 18624 ( 14.0 ) | 15726 ( 57.5 ) |
| cardiac dysrhythmias | No | 97357 ( 60.6 ) | 88376 ( 66.3 ) | 8981 ( 32.8 ) |
|  | Yes | 28961 ( 18.0 ) | 26319 ( 19.7 ) | 2642 ( 9.7 ) |
|  | Missing | 34350 ( 21.4 ) | 18624 ( 14.0 ) | 15726 ( 57.5 ) |
| ischemic heart disease (chronic) | No | 104576 ( 65.1 ) | 94859 ( 71.2 ) | 9717 ( 35.5 ) |
|  | Yes | 21742 ( 13.5 ) | 19836 ( 14.9 ) | 1906 ( 7.0 ) |
|  | Missing | 34350 ( 21.4 ) | 18624 ( 14.0 ) | 15726 ( 57.5 ) |
| hypertension | No | 55208 ( 34.4 ) | 50022 ( 37.5 ) | 5186 ( 19.0 ) |
|  | Yes | 71110 ( 44.3 ) | 64673 ( 48.5 ) | 6437 ( 23.5 ) |
|  | Missing | 34350 ( 21.4 ) | 18624 ( 14.0 ) | 15726 ( 57.5 ) |
| hypotension | No | 115220 ( 71.7 ) | 104572 ( 78.4 ) | 10648 ( 38.9 ) |
|  | Yes | 11098 ( 6.9 ) | 10123 ( 7.6 ) | 975 ( 3.6 ) |
|  | Missing | 34350 ( 21.4 ) | 18624 ( 14.0 ) | 15726 ( 57.5 ) |
| diabetes with complication | No | 124535 ( 77.5 ) | 113068 ( 84.8 ) | 11467 ( 41.9 ) |
|  | Yes | 1783 ( 1.1 ) | 1627 ( 1.2 ) | 156 ( 0.6 ) |
|  | Missing | 34350 ( 21.4 ) | 18624 ( 14.0 ) | 15726 ( 57.5 ) |
| Alzheimer’s or dementia | No | 88637 ( 55.2 ) | 80442 ( 60.3 ) | 8195 ( 30.0 ) |
|  | Yes | 37681 ( 23.5 ) | 34253 ( 25.7 ) | 3428 ( 12.5 ) |
|  | Missing | 34350 ( 21.4 ) | 18624 ( 14.0 ) | 15726 ( 57.5 ) |
| depression | No | 115906 ( 72.1 ) | 105205 ( 78.9 ) | 10701 ( 39.1 ) |
|  | Yes | 10412 ( 6.5 ) | 9490 ( 7.1 ) | 922 ( 3.4 ) |
|  | Missing | 34350 ( 21.4 ) | 18624 ( 14.0 ) | 15726 ( 57.5 ) |
| delirium | No | 115697 ( 72.0 ) | 104877 ( 78.7 ) | 10820 ( 39.6 ) |
|  | Yes | 10621 ( 6.6 ) | 9818 ( 7.4 ) | 803 ( 2.9 ) |
|  | Missing | 34350 ( 21.4 ) | 18624 ( 14.0 ) | 15726 ( 57.5 ) |
| Admitted from location | Own home/sheltered housing | 128941 ( 80.3 ) | 107144 ( 80.4 ) | 21797 ( 79.7 ) |
|  | Nursing care/residential care | 28207 ( 17.6 ) | 23588 ( 17.7 ) | 4619 ( 16.9 ) |
|  | Otherǁ | 3338 ( 2.1 ) | 2567 ( 1.9 ) | 771 ( 2.8 ) |
|  | Missing | 182 ( 0.1 ) | 20 ( 0.0 ) | 162 ( 0.6 ) |

† Comorbidities are identified by the presence of ICD-10 diagnosis codes from the hip fracture care spell, or any admissions in the year prior to the hip fracture care spell.

‡ I – normal healthy individual; II – mild systemic disease that does not limit activity; III – severe systemic disease that limits activity but is not incapacitating; IV-incapacitating systemic disease which is constantly life-threatening; V-moribund -not expected to survive 24 hours with or without surgery.

§ low (less than first quartile), medium (second and third quartile), or high (fourth quartile) volume at admission based on the average annual number of surgeries at the admitting hospital.

ǁ Rehabilitation unit/acute hospital/already in hospital/this hospital site/other hospital site of this trust/other hospital trust

¶ Q1 and Q3: the first and third quarters respectively

# Supplementary File 2

Table S2-1. Characteristics of patients surgically treated for non-pathological first hip fracture overall and by timing of mobilisation and prefracture ambulation

|  |  | **Outdoor ambulation**  **(N=100,983)** | | **Indoor ambulation**  **(N=30,834)** | |
| --- | --- | --- | --- | --- | --- |
|  |  | **Mobilised early (N=82,919)** | **Mobilised late (N=18,064)** | **Mobilised early (N=21,663)** | **Mobilised late (N=9,171)** |
|  |  | **N(%)** | **N(%)** | **N(%)** | **N(%)** |
| Age (years)- median  [Q1-Q3] ¶* |  | 84.0(76-88) | 84 (78-89) | 86 (81-91) | 87 (81-91) |
| Sex* ^**^ | Female | 59970 ( 82.6 ) | 12642 ( 17.4 ) | 16549 ( 71.0 ) | 6755 ( 29.0 ) |
|  | Male | 22949 ( 80.9 ) | 5421 ( 19.1 ) | 5113 ( 67.9 ) | 2416 ( 32.1 ) |
|  | Missing | 0 ( 0.0 ) | 1 ( 100.0 ) | 1 ( 100.0 ) | 0 ( 0.0 ) |
| Ethnicity* | White | 58503 ( 82.6 ) | 12341 ( 17.4 ) | 15855 ( 71.0 ) | 6466 ( 29.0 ) |
|  | Caribbean or African (Black or Black British) or any mixed black background | 99 ( 67.3 ) | 48 ( 32.7 ) | 44 ( 62.0 ) | 27 ( 38.0 ) |
|  | Asian or Asian British or any mixed Asian background | 717 ( 80.7 ) | 172 ( 19.3 ) | 178 ( 66.2 ) | 91 ( 33.8 ) |
|  | Any other Mixed background | 12 ( 66.7 ) | 6 ( 33.3 ) | 6 ( 100.0 ) | 0 ( 0.0 ) |
|  | Missing | 23588 ( 81.1 ) | 5497 ( 18.9 ) | 5580 ( 68.3 ) | 2587 ( 31.7 ) |
| Deprivation* ^**^ | least deprived 10% | 5718 ( 81.3 ) | 1318 ( 18.7 ) | 1911 ( 71.0 ) | 781 ( 29.0 ) |
|  | less deprived 10-20% | 5700 ( 80.9 ) | 1345 ( 19.1 ) | 1767 ( 68.5 ) | 814 ( 31.5 ) |
|  | less deprived 20-30% | 6164 ( 80.9 ) | 1453 ( 19.1 ) | 1946 ( 68.9 ) | 880 ( 31.1 ) |
|  | less deprived 30-40% | 6891 ( 80.9 ) | 1631 ( 19.1 ) | 1879 ( 68.9 ) | 847 ( 31.1 ) |
|  | less deprived 40-50% | 7385 ( 81.8 ) | 1642 ( 18.2 ) | 1936 ( 69.0 ) | 869 ( 31.0 ) |
|  | more deprived 40-50% | 7771 ( 81.7 ) | 1740 ( 18.3 ) | 2064 ( 69.6 ) | 902 ( 30.4 ) |
|  | more deprived 30-40% | 7741 ( 81.9 ) | 1710 ( 18.1 ) | 1976 ( 70.5 ) | 828 ( 29.5 ) |
|  | more deprived 20-30% | 7629 ( 82.6 ) | 1610 ( 17.4 ) | 1874 ( 70.8 ) | 774 ( 29.2 ) |
|  | more deprived 10-20% | 7559 ( 82.5 ) | 1602 ( 17.5 ) | 1963 ( 73.8 ) | 697 ( 26.2 ) |
|  | most deprived 10% | 7458 ( 83.4 ) | 1482 ( 16.6 ) | 1596 ( 70.8 ) | 658 ( 29.2 ) |
|  | Missing | 12903 ( 83.6 ) | 2531 ( 16.4 ) | 2751 ( 71.0 ) | 1121 ( 29.0 ) |
| Fracture type* ^**^ | Intracapsular | 50551 ( 82.8 ) | 10520 ( 17.2 ) | 11886 ( 70.2 ) | 5056 ( 29.8 ) |
|  | Intertrochanteric | 27722 ( 81.7 ) | 6189 ( 18.3 ) | 8609 ( 71.4 ) | 3453 ( 28.6 ) |
|  | Subtrochanteric | 4611 ( 77.4 ) | 1348 ( 22.6 ) | 1161 ( 63.8 ) | 660 ( 36.2 ) |
|  | Missing | 35 ( 83.3 ) | 7 ( 16.7 ) | 7 ( 77.8 ) | 2 ( 22.2 ) |
| Surgery timing* | Within target time | 59848 ( 82.8 ) | 12465 ( 17.2 ) | 15922 ( 71.6 ) | 6308 ( 28.4 ) |
|  | Not within target time | 17757 ( 79.9 ) | 4461 ( 20.1 ) | 4553 ( 65.9 ) | 2351 ( 34.1 ) |
|  | Missing | 5314 ( 82.4 ) | 1138 ( 17.6 ) | 1188 ( 69.9 ) | 512 ( 30.1 ) |
| Procedure type | Internal fixation | 39956 ( 82.3 ) | 8617 ( 17.7 ) | 11005 ( 70.9 ) | 4508 ( 29.1 ) |
|  | Hemiarthroplasty | 33638 ( 80.3 ) | 8278 ( 19.7 ) | 10388 ( 69.6 ) | 4545 ( 30.4 ) |
|  | Total Hip replacement | 9008 ( 89.3 ) | 1084 ( 10.7 ) | 192 ( 74.4 ) | 66 ( 25.6 ) |
|  | Missing/Other | 317 ( 78.9 ) | 85 ( 21.1 ) | 78 ( 60.0 ) | 52 ( 40.0 ) |
| Calendar year of surgery* ^**^ | 2014 | 18905 ( 81.2 ) | 4383 ( 18.8 ) | 5093 ( 68.7 ) | 2321 ( 31.3 ) |
|  | 2015 | 33821 ( 82.8 ) | 7046 ( 17.2 ) | 8469 ( 70.8 ) | 3496 ( 29.2 ) |
|  | 2016 | 30193 ( 82.0 ) | 6635 ( 18.0 ) | 8101 ( 70.7 ) | 3354 ( 29.3 ) |
| Weekday of admission* | Weekday | 55912 ( 81.9 ) | 12351 ( 18.1 ) | 14364 ( 69.8 ) | 6210 ( 30.2 ) |
|  | Weekend | 25846 ( 82.9 ) | 5322 ( 17.1 ) | 6970 ( 71.7 ) | 2747 ( 28.3 ) |
|  | Missing | 1161 ( 74.8 ) | 391 ( 25.2 ) | 329 ( 60.6 ) | 214 ( 39.4 ) |
| Hospital volume§* | High volume | 41730 ( 81.8 ) | 9254 ( 18.2 ) | 11673 ( 70.5 ) | 4878 ( 29.5 ) |
|  | Medium volume | 20497 ( 83.4 ) | 4078 ( 16.6 ) | 4686 ( 71.1 ) | 1908 ( 28.9 ) |
|  | Low volume | 20692 ( 81.4 ) | 4732 ( 18.6 ) | 5304 ( 69.0 ) | 2385 ( 31.0 ) |
| ASA grade‡* | I | 2748 ( 91.4 ) | 259 ( 8.6 ) | 58 ( 72.5 ) | 22 ( 27.5 ) |
|  | II | 28412 ( 87.1 ) | 4196 ( 12.9 ) | 2799 ( 76.6 ) | 857 ( 23.4 ) |
|  | III | 42739 ( 80.5 ) | 10334 ( 19.5 ) | 14128 ( 71.5 ) | 5628 ( 28.5 ) |
|  | IV | 6755 ( 70.8 ) | 2792 ( 29.2 ) | 4247 ( 63.4 ) | 2454 ( 36.6 ) |
|  | V | 104 ( 64.6 ) | 57 ( 35.4 ) | 54 ( 50.9 ) | 52 ( 49.1 ) |
|  | Missing | 2161 ( 83.5 ) | 426 ( 16.5 ) | 377 ( 70.5 ) | 158 ( 29.5 ) |
| Comorbidities†* | heart failure or pulmonary oedema | 6295 ( 74.2 ) | 2192 ( 25.8 ) | 2644 ( 64.9 ) | 1428 ( 35.1 ) |
|  | chronic obstructive pulmonary | 9648 ( 78.4 ) | 2657 ( 21.6 ) | 3166 ( 68.9 ) | 1432 ( 31.1 ) |
|  | ischemic heart (acute) | 6222 ( 78.0 ) | 1759 ( 22.0 ) | 2216 ( 68.5 ) | 1019 ( 31.5 ) |
|  | cardiac dysrhythmias | 14575 ( 78.1 ) | 4095 ( 21.9 ) | 4930 ( 67.4 ) | 2382 ( 32.6 ) |
|  | ischemic heart (chronic) | 10935 ( 78.5 ) | 2996 ( 21.5 ) | 3827 ( 67.7 ) | 1824 ( 32.3 ) |
|  | hypertension | 39863 ( 82.0 ) | 8754 ( 18.0 ) | 10872 ( 70.9 ) | 4464 ( 29.1 ) |
|  | hypotension | 5236 ( 75.7 ) | 1681 ( 24.3 ) | 1997 ( 64.4 ) | 1102 ( 35.6 ) |
|  | diabetes with complication^**^ | 887 ( 78.3 ) | 246 ( 21.7 ) | 317 ( 67.7 ) | 151 ( 32.3 ) |
|  | Alzheimer’s or dementia | 14692 ( 75.9 ) | 4677 ( 24.1 ) | 9678 ( 68.0 ) | 4558 ( 32.0 ) |
|  | depression^**^ | 5231 ( 80.6 ) | 1256 ( 19.4 ) | 2009 ( 69.5 ) | 881 ( 30.5 ) |
|  | delirium | 4524 ( 75.6 ) | 1463 ( 24.4 ) | 2448 ( 66.5 ) | 1233 ( 33.5 ) |
| Admitted from location* | Own home/sheltered housing | 73637 ( 83.4 ) | 14640 ( 16.6 ) | 13039 ( 72.6 ) | 4911 ( 27.4 ) |
|  | Nursing care/residential care | 8148 ( 73.3 ) | 2974 ( 26.7 ) | 8054 ( 67.2 ) | 3925 ( 32.8 ) |
|  | Otherǁ | 1123 ( 71.6 ) | 446 ( 28.4 ) | 569 ( 62.9 ) | 335 ( 37.1 ) |
|  | Missing | 11 ( 73.3 ) | 4 ( 26.7 ) | 1 ( 100.0 ) | 0 ( 0.0 ) |

*P<0.01

† 18,433 cases with missing comorbidity data. Comorbidities are identified by the presence of ICD-10 diagnosis codes from the hip fracture care spell, or any admissions in the year prior to the hip fracture care spell.

‡ I – normal healthy individual; II – mild systemic disease that does not limit activity; III – severe systemic disease that limits activity but is not incapacitating; IV-incapacitating systemic disease which is constantly life-threatening; V-moribund -not expected to survive 24 hours with or without surgery.

§ low (less than first quartile), medium (second and third quartile), or high (fourth quartile) volume at admission based on the average annual number of surgeries at the admitting hospital.

ǁ Rehabilitation unit/acute hospital/already in hospital/this hospital site/other hospital site of this trust/other hospital trust

¶ Q1 and Q3: the first and third quarters respectively

** presence of hypertension or diabetes are not different between early and late mobilised patients among patients with indoor ambulation

Table S2-2. Characteristics of patients surgically treated for non-pathological first hip fracture overall and by timing of mobilization and hypotension*

|  |  | **Without hypotension**  **(N=104,572 )** | | **With hypotension**  **(N=10,123)** | |
| --- | --- | --- | --- | --- | --- |
|  |  | **Mobilised early (N=83,254)** | **Mobilised late (N=21,318)** | **Mobilised early (N=7,298)** | **Mobilised late**  **(N= 2,825)** |
|  |  | **N(%)** | **N(%)** | **N(%)** | **N(%)** |
| Age (years)- median  [Q1-Q3] ¶* |  | 83 [77-89] | 85 [79-90] | 85 [80-89] | 86 [80-90] |
| Sex* | Female | 61093 ( 80.0 ) | 15275 ( 20.0 ) | 4959 ( 72.3 ) | 1904 ( 27.7 ) |
|  | Male | 22161 ( 78.6 ) | 6042 ( 21.4 ) | 2338 ( 71.7 ) | 921 ( 28.3 ) |
|  | Missing | 0 ( 0.0 ) | 1 ( 100.0 ) | 1 ( 100.0 ) | 0 ( 0.0 ) |
| Ethnicity* | White | 68971 ( 80.4 ) | 16765 ( 19.6 ) | 6145 ( 72.6 ) | 2314 ( 27.4 ) |
|  | Caribbean or African (Black or Black British) or any mixed black background | 135 ( 65.9 ) | 70 ( 34.1 ) | 10 ( 62.5 ) | 6 ( 37.5 ) |
|  | Asian or Asian British or any mixed Asian background | 844 ( 77.6 ) | 244 ( 22.4 ) | 61 ( 71.8 ) | 24 ( 28.2 ) |
|  | Any other Mixed background | 18 ( 75.0 ) | 6 ( 25.0 ) |  |  |
|  | Missing | 13286 ( 75.8 ) | 4233 ( 24.2 ) | 1082 ( 69.2 ) | 481 ( 30.8 ) |
| Deprivation* | least deprived 10% | 7129 ( 79.1 ) | 1880 ( 20.9 ) | 606 ( 70.1 ) | 259 ( 29.9 ) |
|  | less deprived 10-20% | 6939 ( 78.1 ) | 1948 ( 21.9 ) | 607 ( 71.0 ) | 248 ( 29.0 ) |
|  | less deprived 20-30% | 7566 ( 78.2 ) | 2107 ( 21.8 ) | 639 ( 70.5 ) | 267 ( 29.5 ) |
|  | less deprived 30-40% | 8127 ( 78.6 ) | 2210 ( 21.4 ) | 737 ( 70.7 ) | 305 ( 29.3 ) |
|  | less deprived 40-50% | 8657 ( 79.3 ) | 2266 ( 20.7 ) | 759 ( 73.6 ) | 272 ( 26.4 ) |
|  | more deprived 40-50% | 9062 ( 79.3 ) | 2363 ( 20.7 ) | 878 ( 73.7 ) | 313 ( 26.3 ) |
|  | more deprived 30-40% | 9058 ( 79.9 ) | 2276 ( 20.1 ) | 763 ( 71.6 ) | 303 ( 28.4 ) |
|  | more deprived 20-30% | 8859 ( 80.7 ) | 2121 ( 19.3 ) | 751 ( 71.2 ) | 304 ( 28.8 ) |
|  | more deprived 10-20% | 8807 ( 81.0 ) | 2072 ( 19.0 ) | 784 ( 74.7 ) | 266 ( 25.3 ) |
|  | most deprived 10% | 8409 ( 81.5 ) | 1906 ( 18.5 ) | 729 ( 73.5 ) | 263 ( 26.5 ) |
|  | Missing | 641 ( 79.1 ) | 169 ( 20.9 ) | 45 ( 64.3 ) | 25 ( 35.7 ) |
| Prefracture ambulation* | Outdoor ambulation | 65309 ( 82.4 ) | 13974 ( 17.6 ) | 5236 ( 75.7 ) | 1681 ( 24.3 ) |
|  | indoor ambulation only | 17065 ( 70.9 ) | 7020 ( 29.1 ) | 1997 ( 64.4 ) | 1102 ( 35.6 ) |
|  | Missing | 880 ( 73.1 ) | 324 ( 26.9 ) | 65 ( 60.7 ) | 42 ( 39.3 ) |
| Fracture type* | Intracapsular | 49677 ( 80.3 ) | 12198 ( 19.7 ) | 4261 ( 72.3 ) | 1633 ( 27.7 ) |
|  | Intertrochanteric | 29009 ( 79.3 ) | 7559 ( 20.7 ) | 2635 ( 72.4 ) | 1003 ( 27.6 ) |
|  | Subtrochanteric | 4529 ( 74.5 ) | 1553 ( 25.5 ) | 399 ( 68.0 ) | 188 ( 32.0 ) |
|  | Missing | 39 ( 83.0 ) | 8 ( 17.0 ) | 3 ( 75.0 ) | 1 ( 25.0 ) |
| Surgery timing* | Within target time | 60507 ( 80.5 ) | 14689 ( 19.5 ) | 5425 ( 73.4 ) | 1968 ( 26.6 ) |
|  | Not within target time | 17575 ( 76.9 ) | 5293 ( 23.1 ) | 1503 ( 67.9 ) | 712 ( 32.1 ) |
|  | Missing | 5172 ( 79.5 ) | 1336 ( 20.5 ) | 370 ( 71.8 ) | 145 ( 28.2 ) |
| Procedure type | Internal fixation | 40678 ( 79.8 ) | 10279 ( 20.2 ) | 3573 ( 73.0 ) | 1322 ( 27.0 ) |
|  | Hemiarthroplasty | 34993 ( 77.7 ) | 10019 ( 22.3 ) | 3321 ( 70.2 ) | 1411 ( 29.8 ) |
|  | Total Hip replacement | 7307 ( 89.0 ) | 902 ( 11.0 ) | 368 ( 81.6 ) | 83 ( 18.4 ) |
|  | Missing/Other | 276 ( 70.1 ) | 118 ( 29.9 ) | 36 ( 80.0 ) | 9 ( 20.0 ) |
| Calendar year of surgery* | 2014 | 22604 ( 78.6 ) | 6137 ( 21.4 ) | 1769 ( 71.8 ) | 695 ( 28.2 ) |
|  | 2015 | 31208 ( 80.4 ) | 7626 ( 19.6 ) | 2706 ( 72.5 ) | 1024 ( 27.5 ) |
|  | 2016 | 29442 ( 79.6 ) | 7555 ( 20.4 ) | 2823 ( 71.9 ) | 1106 ( 28.1 ) |
| Weekday of admission* | Weekday | 56555 ( 79.3 ) | 14733 ( 20.7 ) | 4929 ( 72.1 ) | 1911 ( 27.9 ) |
|  | Weekend | 25513 ( 80.6 ) | 6125 ( 19.4 ) | 2267 ( 73.0 ) | 840 ( 27.0 ) |
|  | Missing | 1186 ( 72.1 ) | 460 ( 27.9 ) | 102 ( 58.0 ) | 74 ( 42.0 ) |
| Hospital volume§* | High volume | 42880 ( 79.4 ) | 11143 ( 20.6 ) | 3705 ( 71.3 ) | 1490 ( 28.7 ) |
|  | Medium volume | 18747 ( 81.2 ) | 4335 ( 18.8 ) | 1750 ( 73.9 ) | 617 ( 26.1 ) |
|  | Low volume | 21627 ( 78.7 ) | 5840 ( 21.3 ) | 1843 ( 72.0 ) | 718 ( 28.0 ) |
| ASA grade‡* | I | 2221 ( 91.0 ) | 220 ( 9.0 ) | 73 ( 83.9 ) | 14 ( 16.1 ) |
|  | II | 24949 ( 86.3 ) | 3974 ( 13.7 ) | 1454 ( 80.3 ) | 357 ( 19.7 ) |
|  | III | 45171 ( 78.4 ) | 12466 ( 21.6 ) | 4437 ( 72.2 ) | 1707 ( 27.8 ) |
|  | IV | 8783 ( 68.1 ) | 4109 ( 31.9 ) | 1114 ( 62.7 ) | 663 ( 37.3 ) |
|  | V | 126 ( 61.5 ) | 79 ( 38.5 ) | 17 ( 54.8 ) | 14 ( 45.2 ) |
|  | Missing | 2004 ( 81.0 ) | 470 ( 19.0 ) | 203 ( 74.4 ) | 70 ( 25.6 ) |
| Comorbidities†* | heart failure or pulmonary edema | 7788 ( 72.0 ) | 3030 ( 28.0 ) | 1268 ( 65.5 ) | 667 ( 34.5 ) |
|  | chronic obstructive pulmonary diseases | 11655 ( 76.5 ) | 3574 ( 23.5 ) | 1302 ( 69.3 ) | 576 ( 30.7 ) |
|  | ischemic heart disease (acute) | 7448 ( 76.2 ) | 2326 ( 23.8 ) | 1090 ( 68.3 ) | 505 ( 31.7 ) |
|  | cardiac dysrhythmias | 17400 ( 75.9 ) | 5521 ( 24.1 ) | 2321 ( 68.3 ) | 1077 ( 31.7 ) |
|  | ischemic heart disease (chronic) | 13132 ( 76.1 ) | 4123 ( 23.9 ) | 1801 ( 69.8 ) | 780 ( 30.2 ) |
|  | hypertension** | 46779 ( 79.9 ) | 11801 ( 20.1 ) | 4473 ( 73.4 ) | 1620 ( 26.6 ) |
|  | diabetes with complication | 1065 ( 74.9 ) | 356 ( 25.1 ) | 156 ( 75.7 ) | 50 ( 24.3 ) |
|  | Alzheimer’s or dementia | 22158 ( 73.1 ) | 8161 ( 26.9 ) | 2652 ( 67.4 ) | 1282 ( 32.6 ) |
|  | depression** | 6436 ( 77.7 ) | 1847 ( 22.3 ) | 882 ( 73.1 ) | 325 ( 26.9 ) |
|  | delirium | 6056 ( 73.1 ) | 2234 ( 26.9 ) | 1016 ( 66.5 ) | 512 ( 33.5 ) |
| Admitted from location* | Own home/sheltered housing | 68619 ( 81.8 ) | 15235 ( 18.2 ) | 5662 ( 74.7 ) | 1922 ( 25.3 ) |
|  | Nursing care/residential care | 13162 ( 70.8 ) | 5427 ( 29.2 ) | 1441 ( 64.0 ) | 809 ( 36.0 ) |
|  | Otherǁ | 1458 ( 69.1 ) | 652 ( 30.9 ) | 195 ( 67.5 ) | 94 ( 32.5 ) |
|  | Missing | 15 ( 78.9 ) | 4 ( 21.1 ) | 0 | 0 |

*<0.01

† Comorbidities are identified by the presence of ICD-10 diagnosis codes from the hip fracture care spell, or any admissions in the year prior to the hip fracture care spell.

‡ I – normal healthy individual; II – mild systemic disease that does not limit activity; III – severe systemic disease that limits activity but is not incapacitating; IV-incapacitating systemic disease which is constantly life-threatening; V-moribund -not expected to survive 24 hours with or without surgery.

§ low (less than first quartile), medium (second and third quartile), or high (fourth quartile) volume at admission based on the average annual number of surgeries at the admitting hospital.

ǁ Rehabilitation unit/acute hospital/already in hospital/this hospital site/other hospital site of this trust/other hospital trust

¶ Q1 and Q3: the first and third quarters respectively

** Sex, deprivation, fracture type, calendar year of surgery, hospital volume, presence of hypertension or diabetes are not different between early and late mobilised patients among patients with hypotension.

Table S2-3. Characteristics of patients surgically treated for non-pathological first hip fracture overall and by timing of mobilization and dementia

|  |  | **Without dementia**  **(N=80,442)** | | **With dementia**  **(N=34,253)** | |
| --- | --- | --- | --- | --- | --- |
|  |  | **Mobilised early (N=65,742)** | **Mobilised late (N=14,700)** | **Mobilised early (N= 24,810 )** | **Mobilised late (N= 19,443)** |
|  |  | **N(%)** | **N(%)** | **N(%)** | **N(%)** |
| Age (years)- median  [Q1-Q3] ¶* |  | 82 [75-88] | 84 [77-89] | 86 [82-91] | 87 [82-91] |
| Sex* | Female | 47607 ( 82.1 ) | 10395 ( 17.9 ) | 18445 ( 73.1 ) | 6784 ( 26.9 ) |
|  | Male | 18134 ( 80.8 ) | 4305 ( 19.2 ) | 6365 ( 70.5 ) | 2658 ( 29.5 ) |
|  | Missing | 1 ( 100.0 ) | 0 ( 0.0 ) | 0 ( 0.0 ) | 1 ( 100.0 ) |
| Ethnicity* | White | 54279 ( 82.6 ) | 11455 ( 17.4 ) | 20837 ( 73.2 ) | 7624 ( 26.8 ) |
|  | Caribbean or African (Black or Black British) or any mixed black background | 94 ( 67.1 ) | 46 ( 32.9 ) | 51 ( 63.0 ) | 30 ( 37.0 ) |
|  | Asian or Asian British or any mixed Asian background | 741 ( 80.3 ) | 182 ( 19.7 ) | 164 ( 65.6 ) | 86 ( 34.4 ) |
|  | Any other Mixed background | 15 ( 71.4 ) | 6 ( 28.6 ) | 3 ( 100.0 ) | 0 ( 0.0 ) |
|  | Missing | 10613 ( 77.9 ) | 3011 ( 22.1 ) | 3755 ( 68.8 ) | 1703 ( 31.2 ) |
| Deprivation* | least deprived 10% | 5600 ( 81.2 ) | 1295 ( 18.8 ) | 2135 ( 71.7 ) | 844 ( 28.3 ) |
|  | less deprived 10-20% | 5385 ( 80.3 ) | 1321 ( 19.7 ) | 2161 ( 71.2 ) | 875 ( 28.8 ) |
|  | less deprived 20-30% | 5816 ( 80.2 ) | 1433 ( 19.8 ) | 2389 ( 71.7 ) | 941 ( 28.3 ) |
|  | less deprived 30-40% | 6362 ( 80.9 ) | 1500 ( 19.1 ) | 2502 ( 71.1 ) | 1015 ( 28.9 ) |
|  | less deprived 40-50% | 6866 ( 81.7 ) | 1542 ( 18.3 ) | 2550 ( 71.9 ) | 996 ( 28.1 ) |
|  | more deprived 40-50% | 7183 ( 81.7 ) | 1610 ( 18.3 ) | 2757 ( 72.1 ) | 1066 ( 27.9 ) |
|  | more deprived 30-40% | 7164 ( 82.1 ) | 1567 ( 17.9 ) | 2657 ( 72.4 ) | 1012 ( 27.6 ) |
|  | more deprived 20-30% | 7071 ( 82.6 ) | 1490 ( 17.4 ) | 2539 ( 73.1 ) | 935 ( 26.9 ) |
|  | more deprived 10-20% | 7007 ( 82.6 ) | 1476 ( 17.4 ) | 2584 ( 75.0 ) | 862 ( 25.0 ) |
|  | most deprived 10% | 6771 ( 83.5 ) | 1342 ( 16.5 ) | 2367 ( 74.1 ) | 827 ( 25.9 ) |
|  | Missing | 517 ( 80.7 ) | 124 ( 19.3 ) | 169 ( 70.7 ) | 70 ( 29.3 ) |
| Prefracture ambulation* | Outdoor ambulation | 55853 ( 83.6 ) | 10978 ( 16.4 ) | 14692 ( 75.9 ) | 4677 ( 24.1 ) |
|  | indoor ambulation only | 9384 ( 72.5 ) | 3564 ( 27.5 ) | 9678 ( 68.0 ) | 4558 ( 32.0 ) |
|  | Missing | 505 ( 76.2 ) | 158 ( 23.8 ) | 440 ( 67.9 ) | 208 ( 32.1 ) |
| Fracture type* | Intracapsular | 39733 ( 82.6 ) | 8345 ( 17.4 ) | 14205 ( 72.1 ) | 5486 ( 27.9 ) |
|  | Intertrochanteric | 22225 ( 81.2 ) | 5146 ( 18.8 ) | 9419 ( 73.4 ) | 3416 ( 26.6 ) |
|  | Subtrochanteric | 3758 ( 75.7 ) | 1205 ( 24.3 ) | 1170 ( 68.6 ) | 536 ( 31.4 ) |
|  | Missing | 26 ( 86.7 ) | 4 ( 13.3 ) | 16 ( 76.2 ) | 5 ( 23.8 ) |
| Surgery timing* | Within target time | 47522 ( 82.8 ) | 9880 ( 17.2 ) | 18410 ( 73.1 ) | 6777 ( 26.9 ) |
|  | Not within target time | 14113 ( 78.5 ) | 3855 ( 21.5 ) | 4965 ( 69.8 ) | 2150 ( 30.2 ) |
|  | Missing | 4107 ( 81.0 ) | 965 ( 19.0 ) | 1435 ( 73.6 ) | 516 ( 26.4 ) |
| Procedure type* | Internal fixation | 31944 ( 81.7 ) | 7164 ( 18.3 ) | 12307 ( 73.5 ) | 4437 ( 26.5 ) |
|  | Hemiarthroplasty | 26166 ( 80.0 ) | 6549 ( 20.0 ) | 12148 ( 71.3 ) | 4881 ( 28.7 ) |
|  | Total Hip replacement | 7398 ( 89.0 ) | 910 ( 11.0 ) | 277 ( 78.7 ) | 75 ( 21.3 ) |
|  | Missing/Other | 234 ( 75.2 ) | 77 ( 24.8 ) | 78 ( 60.9 ) | 50 ( 39.1 ) |
| Calendar year of surgery* | 2014 | 17848 ( 80.6 ) | 4291 ( 19.4 ) | 6525 ( 72.0 ) | 2541 ( 28.0 ) |
|  | 2015 | 24670 ( 82.2 ) | 5337 ( 17.8 ) | 9244 ( 73.6 ) | 3313 ( 26.4 ) |
|  | 2016 | 23224 ( 82.1 ) | 5072 ( 17.9 ) | 9041 ( 71.6 ) | 3589 ( 28.4 ) |
| Weekday of admission* | Weekday | 44938 ( 81.4 ) | 10280 ( 18.6 ) | 16546 ( 72.2 ) | 6364 ( 27.8 ) |
|  | Weekend | 19856 ( 82.9 ) | 4082 ( 17.1 ) | 7924 ( 73.3 ) | 2883 ( 26.7 ) |
|  | Missing | 948 ( 73.7 ) | 338 ( 26.3 ) | 340 ( 63.4 ) | 196 ( 36.6 ) |
| Hospital volume§* | High volume | 33598 ( 81.5 ) | 7645 ( 18.5 ) | 12987 ( 72.3 ) | 4988 ( 27.7 ) |
|  | Medium volume | 14810 ( 83.2 ) | 2998 ( 16.8 ) | 5687 ( 74.4 ) | 1954 ( 25.6 ) |
|  | Low volume | 17334 ( 81.0 ) | 4057 ( 19.0 ) | 6136 ( 71.0 ) | 2501 ( 29.0 ) |
| ASA grade‡* | I | 2196 ( 91.0 ) | 216 ( 9.0 ) | 98 ( 84.5 ) | 18 ( 15.5 ) |
|  | II | 23058 ( 87.1 ) | 3427 ( 12.9 ) | 3345 ( 78.7 ) | 904 ( 21.3 ) |
|  | III | 33377 ( 80.2 ) | 8215 ( 19.8 ) | 16231 ( 73.1 ) | 5958 ( 26.9 ) |
|  | IV | 5496 ( 69.1 ) | 2454 ( 30.9 ) | 4401 ( 65.5 ) | 2318 ( 34.5 ) |
|  | V | 75 ( 62.5 ) | 45 ( 37.5 ) | 68 ( 58.6 ) | 48 ( 41.4 ) |
|  | Missing | 1540 ( 81.8 ) | 343 ( 18.2 ) | 667 ( 77.2 ) | 197 ( 22.8 ) |
| Comorbidities†* | heart failure or pulmonary edema | 6116 ( 73.0 ) | 2263 ( 27.0 ) | 2940 ( 67.2 ) | 1434 ( 32.8 ) |
|  | chronic obstructive pulmonary diseases | 9753 ( 77.6 ) | 2810 ( 22.4 ) | 3204 ( 70.5 ) | 1340 ( 29.5 ) |
|  | ischemic heart disease (acute) | 5966 ( 77.2 ) | 1764 ( 22.8 ) | 2572 ( 70.7 ) | 1067 ( 29.3 ) |
|  | cardiac dysrhythmias | 13373 ( 77.4 ) | 3904 ( 22.6 ) | 6348 ( 70.2 ) | 2694 ( 29.8 ) |
|  | ischemic heart disease (chronic) | 10384 ( 77.8 ) | 2968 ( 22.2 ) | 4549 ( 70.2 ) | 1935 ( 29.8 ) |
|  | Hypertension** | 37592 ( 81.6 ) | 8454 ( 18.4 ) | 13660 ( 73.3 ) | 4967 ( 26.7 ) |
|  | hypotension | 4646 ( 75.1 ) | 1543 ( 24.9 ) | 2652 ( 67.4 ) | 1282 ( 32.6 ) |
|  | diabetes with complication** | 902 ( 77.1 ) | 268 ( 22.9 ) | 319 ( 69.8 ) | 138 ( 30.2 ) |
|  | depression** | 4595 ( 80.6 ) | 1105 ( 19.4 ) | 2723 ( 71.8 ) | 1067 ( 28.2 ) |
|  | Delirium** | 65742 ( 81.7 ) | 14700 ( 18.3 ) | 7072 (72.0 ) | 2746 (28.0 ) |
| Admitted from location* | Own home/sheltered housing | 61055 ( 82.5 ) | 12907 ( 17.5 ) | 13226 ( 75.7 ) | 4250 ( 24.3 ) |
|  | Nursing care/residential care | 3758 ( 72.8 ) | 1406 ( 27.2 ) | 10845 ( 69.2 ) | 4830 ( 30.8 ) |
|  | Other | 916 ( 70.5 ) | 384 ( 29.5 ) | 737 ( 67.1 ) | 362 ( 32.9 ) |
|  | Missing | 13 ( 81.2 ) | 3 ( 18.8 ) | 2 ( 66.7 ) | 1 ( 33.3 ) |

*<0.01

† Comorbidities are identified by the presence of ICD-10 diagnosis codes from the hip fracture care spell, or any admissions in the year prior to the hip fracture care spell.

‡ I – normal healthy individual; II – mild systemic disease that does not limit activity; III – severe systemic disease that limits activity but is not incapacitating; IV-incapacitating systemic disease which is constantly life-threatening; V-moribund -not expected to survive 24 hours with or without surgery.

§ low (less than first quartile), medium (second and third quartile), or high (fourth quartile) volume at admission based on the average annual number of surgeries at the admitting hospital.

ǁ Rehabilitation unit/acute hospital/already in hospital/this hospital site/other hospital site of this trust/other hospital trust

¶ Q1 and Q3: the first and third quarters respectively

** presence of hypertension is not different between early and late mobilised patients among patients without dementia. Presence of delirium in patients without dementia. Presence of diabetes, depression and delirium are not different between early and late mobilised patients among patients with dementia.

Table S2-4. Characteristics of patients surgically treated for non-pathological first hip fracture overall and by timing of mobilisation and delirium

|  |  | **Without delirium**  **(N=104,877)** | | **With delirium**  **(N=9,818)** | |
| --- | --- | --- | --- | --- | --- |
|  |  | **Mobilised early (N=83,480)** | **Mobilised late (N=21,397)** | **Mobilised early (N=7,072)** | **Mobilised late (N=2,746)** |
|  |  | **N(%)** | **N(%)** | **N(%)** | **N(%)** |
| Age (years)- median  [Q1-Q3¶]* |  | 83 [77-89] | 85 [79-90] | 86 [81-91] | 87 [82-91] |
| Sex* ^**^ | Female | 61228 ( 79.9 ) | 15358 ( 20.1 ) | 4824 ( 72.6 ) | 1821 ( 27.4 ) |
|  | Male | 22251 ( 78.7 ) | 6038 ( 21.3 ) | 2248 ( 70.8 ) | 925 ( 29.2 ) |
|  | Missing | 1 ( 50.0 ) | 1 ( 50.0 ) | 0 (0.0) | 0 (0.0) |
| Ethnicity* ^**^ | White | 69066 ( 80.5 ) | 16754 ( 19.5 ) | 6050 ( 72.2 ) | 2325 ( 27.8 ) |
|  | Caribbean or African (Black or Black British) or any mixed black background | 132 ( 65.7 ) | 69 ( 34.3 ) | 13 ( 65.0 ) | 7 ( 35.0 ) |
|  | Asian or Asian British or any mixed Asian background | 862 ( 78.1 ) | 242 ( 21.9 ) | 43 ( 62.3 ) | 26 ( 37.7 ) |
|  | Any other Mixed background | 17 ( 73.9 ) | 6 ( 26.1 ) | 1 ( 100.0 ) | 0 ( 0.0 ) |
|  | Missing | 13403 ( 75.6 ) | 4326 ( 24.4 ) | 965 ( 71.3 ) | 388 ( 28.7 ) |
| Deprivation* | least deprived 10% | 7137 ( 78.9 ) | 1904 ( 21.1 ) | 598 ( 71.8 ) | 235 ( 28.2 ) |
|  | less deprived 10-20% | 6933 ( 78.2 ) | 1930 ( 21.8 ) | 613 ( 69.7 ) | 266 ( 30.3 ) |
|  | less deprived 20-30% | 7526 ( 78.1 ) | 2114 ( 21.9 ) | 679 ( 72.3 ) | 260 ( 27.7 ) |
|  | less deprived 30-40% | 8157 ( 78.6 ) | 2226 ( 21.4 ) | 707 ( 71.0 ) | 289 ( 29.0 ) |
|  | less deprived 40-50% | 8710 ( 79.6 ) | 2239 ( 20.4 ) | 706 ( 70.2 ) | 299 ( 29.8 ) |
|  | more deprived 40-50% | 9199 ( 79.6 ) | 2359 ( 20.4 ) | 741 ( 70.0 ) | 317 ( 30.0 ) |
|  | more deprived 30-40% | 9055 ( 79.9 ) | 2272 ( 20.1 ) | 766 ( 71.4 ) | 307 ( 28.6 ) |
|  | more deprived 20-30% | 8908 ( 80.4 ) | 2165 ( 19.6 ) | 702 ( 73.0 ) | 260 ( 27.0 ) |
|  | more deprived 10-20% | 8804 ( 80.8 ) | 2091 ( 19.2 ) | 787 ( 76.1 ) | 247 ( 23.9 ) |
|  | most deprived 10% | 8415 ( 81.4 ) | 1927 ( 18.6 ) | 723 ( 74.9 ) | 242 ( 25.1 ) |
|  | Missing | 636 ( 78.9 ) | 170 ( 21.1 ) | 50 ( 67.6 ) | 24 ( 32.4 ) |
| Prefracture ambulation* | Outdoor ambulation | 66021 ( 82.3 ) | 14192 ( 17.7 ) | 4524 ( 75.6 ) | 1463 ( 24.4 ) |
|  | indoor ambulation only | 16614 ( 70.7 ) | 6889 ( 29.3 ) | 2448 ( 66.5 ) | 1233 ( 33.5 ) |
|  | Missing | 845 ( 72.8 ) | 316 ( 27.2 ) | 100 ( 66.7 ) | 50 ( 33.3 ) |
| Hip fracture type* | Intracapsular | 49826 ( 80.2 ) | 12267 ( 19.8 ) | 4112 ( 72.4 ) | 1564 ( 27.6 ) |
|  | Intertrochanteric | 29014 ( 79.4 ) | 7546 ( 20.6 ) | 2630 ( 72.1 ) | 1016 ( 27.9 ) |
|  | Subtrochanteric | 4600 ( 74.5 ) | 1576 ( 25.5 ) | 328 ( 66.5 ) | 165 ( 33.5 ) |
|  | Missing | 40 ( 83.3 ) | 8 ( 16.7 ) | 2 ( 66.7 ) | 1 ( 33.3 ) |
| Surgery timing* | Within target time | 60615 ( 80.5 ) | 14677 ( 19.5 ) | 5317 ( 72.9 ) | 1980 ( 27.1 ) |
|  | Not within target time | 17659 ( 76.8 ) | 5346 ( 23.2 ) | 1419 ( 68.3 ) | 659 ( 31.7 ) |
|  | Missing | 5206 ( 79.1 ) | 1374 ( 20.9 ) | 336 ( 75.8 ) | 107 ( 24.2 ) |
| Procedure type* | Internal fixation | 40830 ( 79.9 ) | 10288 ( 20.1 ) | 3421 ( 72.3 ) | 1313 ( 27.7 ) |
|  | Hemiarthroplasty | 34839 ( 77.6 ) | 10050 ( 22.4 ) | 3475 ( 71.6 ) | 1380 ( 28.4 ) |
|  | Total Hip replacement | 7510 ( 88.8 ) | 946 ( 11.2 ) | 165 ( 80.9 ) | 39 ( 19.1 ) |
|  | Missing/Other | 301 ( 72.7 ) | 113 ( 27.3 ) | 11 ( 44.0 ) | 14 ( 56.0 ) |
| Calendar year of surgery* | 2014 | 22872 ( 78.5 ) | 6253 ( 21.5 ) | 1501 ( 72.2 ) | 579 ( 27.8 ) |
|  | 2015 | 31309 ( 80.3 ) | 7705 ( 19.7 ) | 2605 ( 73.4 ) | 945 ( 26.6 ) |
|  | 2016 | 29299 ( 79.8 ) | 7439 ( 20.2 ) | 2966 ( 70.8 ) | 1222 ( 29.2 ) |
| Weekday of admission* | Weekday | 56788 ( 79.3 ) | 14826 ( 20.7 ) | 4696 ( 72.1 ) | 1818 ( 27.9 ) |
|  | Weekend | 25504 ( 80.7 ) | 6104 ( 19.3 ) | 2276 ( 72.6 ) | 861 ( 27.4 ) |
|  | Missing | 1188 ( 71.8 ) | 467 ( 28.2 ) | 100 ( 59.9 ) | 67 ( 40.1 ) |
| Hospital volume§* | High volume | 42777 ( 79.2 ) | 11245 ( 20.8 ) | 3808 ( 73.3 ) | 1388 ( 26.7 ) |
|  | Medium volume | 18789 ( 81.4 ) | 4293 ( 18.6 ) | 1708 ( 72.2 ) | 659 ( 27.8 ) |
|  | Low volume | 21914 ( 78.9 ) | 5859 ( 21.1 ) | 1556 ( 69.0 ) | 699 ( 31.0 ) |
| ASA grade‡* | I | 2247 ( 90.8 ) | 229 ( 9.2 ) | 47 ( 90.4 ) | 5 ( 9.6 ) |
|  | II | 25308 ( 86.3 ) | 4025 ( 13.7 ) | 1095 ( 78.2 ) | 306 ( 21.8 ) |
|  | III | 45159 ( 78.2 ) | 12565 ( 21.8 ) | 4449 ( 73.5 ) | 1608 ( 26.5 ) |
|  | IV | 8615 ( 68.2 ) | 4026 ( 31.8 ) | 1282 ( 63.2 ) | 746 ( 36.8 ) |
|  | V | 129 ( 61.7 ) | 80 ( 38.3 ) | 14 ( 51.9 ) | 13 ( 48.1 ) |
|  | Missing | 2022 ( 81.1 ) | 472 ( 18.9 ) | 185 ( 73.1 ) | 68 ( 26.9 ) |
| Comorbidities†* | heart failure or pulmonary edema | 7970 ( 71.9 ) | 3121 ( 28.1 ) | 1086 ( 65.3 ) | 576 ( 34.7 ) |
|  | chronic obstructive pulmonary diseases | 11811 ( 76.4 ) | 3646 ( 23.6 ) | 1146 ( 69.5 ) | 504 ( 30.5 ) |
|  | ischemic heart disease (acute) | 7669 ( 75.8 ) | 2448 ( 24.2 ) | 869 ( 69.4 ) | 383 ( 30.6 ) |
|  | cardiac dysrhythmias | 17522 ( 75.6 ) | 5663 ( 24.4 ) | 2199 ( 70.2 ) | 935 ( 29.8 ) |
|  | ischemic heart disease (chronic) | 13423 ( 76.1 ) | 4209 ( 23.9 ) | 1510 ( 68.5 ) | 694 ( 31.5 ) |
|  | hypertension^**^ | 47069 ( 79.9 ) | 11841 ( 20.1 ) | 4183 ( 72.6 ) | 1580 ( 27.4 ) |
|  | hypotension | 6282 ( 73.1 ) | 2313 ( 26.9 ) | 1016 ( 66.5 ) | 512 ( 33.5 ) |
|  | diabetes with complication^**^ | 1081 ( 75.8 ) | 345 ( 24.2 ) | 140 ( 69.7 ) | 61 ( 30.3 ) |
|  | Alzheimer’s or dementia | 17738 ( 72.6 ) | 6697 ( 27.4 ) | 7072 ( 72.0 ) | 2746 ( 28.0 ) |
|  | depression^**^ | 6473 ( 77.9 ) | 1836 ( 22.1 ) | 845 ( 71.5 ) | 336 ( 28.5 ) |
| Admitted from location* | Own home/sheltered housing | 69518 ( 81.7 ) | 15525 ( 18.3 ) | 4763 ( 74.5 ) | 1632 ( 25.5 ) |
|  | Nursing care/residential care | 12563 ( 70.5 ) | 5255 ( 29.5 ) | 2040 ( 67.5 ) | 981 ( 32.5 ) |
|  | Other | 1385 ( 69.3 ) | 613 ( 30.7 ) | 268 ( 66.8 ) | 133 ( 33.2 ) |
|  | Missing | 14 ( 77.8 ) | 4 ( 22.2 ) | 1 ( 100.0 ) | 0 ( 0.0 ) |

*<0.01

† Comorbidities are identified by the presence of ICD-10 diagnosis codes from the hip fracture care spell, or any admissions in the year prior to the hip fracture care spell.

‡ I – normal healthy individual; II – mild systemic disease that does not limit activity; III – severe systemic disease that limits activity but is not incapacitating; IV-incapacitating systemic disease which is constantly life-threatening; V-moribund -not expected to survive 24 hours with or without surgery.

§ low (less than first quartile), medium (second and third quartile), or high (fourth quartile) volume at admission based on the average annual number of surgeries at the admitting hospital.

ǁ Rehabilitation unit/acute hospital/already in hospital/this hospital site/other hospital site of this trust/other hospital trust

¶ Q1 and Q3: the first and third quarters respectively

** Age, sex, ethnicity, presence of hypertension, diabetes and depression are not different between early and late mobilised patients among patients with delirium.

Table S2-5. Characteristics of patients surgically treated for non-pathological first hip fracture overall and by timing of mobilisation and prefracture residence

|  |  | **Own home/sheltered housing**  **(N=107,144)** | | **Nursing care/residential care**  **(N=23,588)** | |
| --- | --- | --- | --- | --- | --- |
|  |  | **Mobilised early (N=87,365)** | **Mobilised late (N=19,779)** | **Mobilised early (N=16,523)** | **Mobilised late (N=7,065)** |
| Age (years)- median  [Q1-Q3¶]* |  | 83 [76-88] | 84 [78-89] | 87 [83-92] | 87 [82-92] |
|  | Missing | 163 ( 80.3 ) | 40 ( 19.7 ) | 13 ( 72.2 ) | 5 ( 27.8 ) |
| Sex* | Female | 63417 ( 81.9 ) | 13980 ( 18.1 ) | 12775 ( 71.0 ) | 5225 ( 29.0 ) |
|  | Male | 23947 ( 80.5 ) | 5799 ( 19.5 ) | 3748 ( 67.1 ) | 1839 ( 32.9 ) |
|  | Missing | 1 ( 100.0 ) | 0 ( 0.0 ) | 0 ( 0.0 ) | 1 ( 100.0 ) |
| Ethnicity* | White | 61381 ( 82.1 ) | 13387 ( 17.9 ) | 12346 ( 70.8 ) | 5082 ( 29.2 ) |
|  | Caribbean or African (Black or Black British) or any mixed black background | 114 ( 68.7 ) | 52 ( 31.3 ) | 26 ( 54.2 ) | 22 ( 45.8 ) |
|  | Asian or Asian British or any mixed Asian background | 849 ( 78.3 ) | 235 ( 21.7 ) | 40 ( 61.5 ) | 25 ( 38.5 ) |
|  | Any other Mixed background | 17 ( 73.9 ) | 6 ( 26.1 ) | 1 ( 100.0 ) | 0 ( 0.0 ) |
|  | Missing | 25004 ( 80.4 ) | 6099 ( 19.6 ) | 4110 ( 68.0 ) | 1936 ( 32.0 ) |
| Deprivation* ** | least deprived 10% | 6303 ( 80.7 ) | 1509 ( 19.3 ) | 1259 ( 68.9 ) | 567 ( 31.1 ) |
|  | less deprived 10-20% | 6084 ( 79.7 ) | 1547 ( 20.3 ) | 1289 ( 69.9 ) | 555 ( 30.1 ) |
|  | less deprived 20-30% | 6594 ( 80.3 ) | 1615 ( 19.7 ) | 1456 ( 68.3 ) | 675 ( 31.7 ) |
|  | less deprived 30-40% | 7255 ( 80.1 ) | 1804 ( 19.9 ) | 1463 ( 70.1 ) | 623 ( 29.9 ) |
|  | less deprived 40-50% | 7741 ( 81.0 ) | 1818 ( 19.0 ) | 1491 ( 69.5 ) | 653 ( 30.5 ) |
|  | more deprived 40-50% | 8104 ( 81.0 ) | 1900 ( 19.0 ) | 1636 ( 69.4 ) | 721 ( 30.6 ) |
|  | more deprived 30-40% | 8068 ( 81.1 ) | 1875 ( 18.9 ) | 1582 ( 72.0 ) | 615 ( 28.0 ) |
|  | more deprived 20-30% | 7921 ( 82.6 ) | 1671 ( 17.4 ) | 1532 ( 69.2 ) | 682 ( 30.8 ) |
|  | more deprived 10-20% | 7953 ( 82.4 ) | 1693 ( 17.6 ) | 1489 ( 72.1 ) | 575 ( 27.9 ) |
|  | most deprived 10% | 7704 ( 82.9 ) | 1593 ( 17.1 ) | 1293 ( 71.3 ) | 520 ( 28.7 ) |
|  | Missing | 13638 ( 83.2 ) | 2754 ( 16.8 ) | 2033 ( 69.8 ) | 879 ( 30.2 ) |
| Prefracture ambulation* | Outdoor ambulation | 73637 ( 83.4 ) | 14640 ( 16.6 ) | 8148 ( 73.3 ) | 2974 ( 26.7 ) |
|  | indoor ambulation only | 13039 ( 72.6 ) | 4911 ( 27.4 ) | 8054 ( 67.2 ) | 3925 ( 32.8 ) |
|  | Missing | 689 ( 75.1 ) | 228 ( 24.9 ) | 321 ( 65.9 ) | 166 ( 34.1 ) |
| Hip fracture type* | Intracapsular | 52715 ( 82.4 ) | 11241 ( 17.6 ) | 9269 ( 69.3 ) | 4100 ( 30.7 ) |
|  | Intertrochanteric | 29619 ( 81.0 ) | 6929 ( 19.0 ) | 6477 ( 71.6 ) | 2572 ( 28.4 ) |
|  | Subtrochanteric | 4992 ( 75.7 ) | 1600 ( 24.3 ) | 770 ( 66.3 ) | 391 ( 33.7 ) |
|  | Missing | 39 ( 81.2 ) | 9 ( 18.8 ) | 7 ( 77.8 ) | 2 ( 22.2 ) |
| Surgery timing* | Within target time | 63029 ( 82.5 ) | 13375 ( 17.5 ) | 12286 ( 70.4 ) | 5161 ( 29.6 ) |
|  | Not within target time | 18858 ( 78.5 ) | 5165 ( 21.5 ) | 3278 ( 68.1 ) | 1536 ( 31.9 ) |
|  | Missing | 5478 ( 81.6 ) | 1239 ( 18.4 ) | 959 ( 72.3 ) | 368 ( 27.7 ) |
| Procedure type* | Internal fixation | 42212 ( 81.5 ) | 9597 ( 18.5 ) | 8387 ( 71.6 ) | 3333 ( 28.4 ) |
|  | Hemiarthroplasty | 35716 ( 79.9 ) | 8979 ( 20.1 ) | 7972 ( 68.5 ) | 3660 ( 31.5 ) |
|  | Total Hip replacement | 9108 ( 89.2 ) | 1103 ( 10.8 ) | 98 ( 73.7 ) | 35 ( 26.3 ) |
|  | Missing/Other | 329 ( 76.7 ) | 100 ( 23.3 ) | 66 ( 64.1 ) | 37 ( 35.9 ) |
| Calendar year of surgery* | 2014 | 19548 ( 80.5 ) | 4726 ( 19.5 ) | 4090 ( 69.9 ) | 1762 ( 30.1 ) |
|  | 2015 | 35040 ( 82.3 ) | 7530 ( 17.7 ) | 6678 ( 71.1 ) | 2710 ( 28.9 ) |
|  | 2016 | 32777 ( 81.3 ) | 7523 ( 18.7 ) | 5755 ( 68.9 ) | 2593 ( 31.1 ) |
| Weekday of admission* | Weekday | 58926 ( 81.2 ) | 13653 ( 18.8 ) | 10813 ( 69.9 ) | 4657 ( 30.1 ) |
|  | Weekend | 27205 ( 82.7 ) | 5694 ( 17.3 ) | 5493 ( 70.8 ) | 2269 ( 29.2 ) |
|  | Missing | 1234 ( 74.1 ) | 432 ( 25.9 ) | 217 ( 61.0 ) | 139 ( 39.0 ) |
| Hospital volume§* | High volume | 44543 ( 81.4 ) | 10176 ( 18.6 ) | 8612 ( 69.5 ) | 3771 ( 30.5 ) |
|  | Medium volume | 20954 ( 82.9 ) | 4315 ( 17.1 ) | 4001 ( 71.9 ) | 1567 ( 28.1 ) |
|  | Low volume | 21868 ( 80.5 ) | 5288 ( 19.5 ) | 3910 ( 69.4 ) | 1727 ( 30.6 ) |
| ASA grade‡* | I | 2775 ( 91.2 ) | 269 ( 8.8 ) | 42 ( 80.8 ) | 10 ( 19.2 ) |
|  | II | 29383 ( 86.8 ) | 4479 ( 13.2 ) | 1797 ( 76.7 ) | 545 ( 23.3 ) |
|  | III | 45611 ( 80.2 ) | 11266 ( 19.8 ) | 10804 ( 70.7 ) | 4477 ( 29.3 ) |
|  | IV | 7372 ( 69.1 ) | 3296 ( 30.9 ) | 3377 ( 65.2 ) | 1800 ( 34.8 ) |
|  | V | 114 ( 63.7 ) | 65 ( 36.3 ) | 44 ( 54.3 ) | 37 ( 45.7 ) |
|  | Missing | 2110 ( 83.9 ) | 404 ( 16.1 ) | 459 ( 70.1 ) | 196 ( 29.9 ) |
|  | heart failure or pulmonary edema | 7003 ( 72.5 ) | 2650 ( 27.5 ) | 1774 ( 66.9 ) | 877 ( 33.1 ) |
| Comorbidities† | chronic obstructive pulmonary diseases** | 10861 ( 77.0 ) | 3241 ( 23.0 ) | 1761 ( 69.9 ) | 759 ( 30.1 ) |
|  | ischemic heart disease (acute) ** | 6797 ( 77.0 ) | 2028 ( 23.0 ) | 1521 ( 68.8 ) | 691 ( 31.2 ) |
|  | cardiac dysrhythmias** | 15526 ( 76.9 ) | 4671 ( 23.1 ) | 3684 ( 69.0 ) | 1656 ( 31.0 ) |
|  | ischemic heart disease (chronic) †† | 11914 ( 77.4 ) | 3478 ( 22.6 ) | 2617 ( 68.2 ) | 1223 ( 31.8 ) |
|  | hypertension* | 42625 ( 81.2 ) | 9859 ( 18.8 ) | 7670 ( 71.0 ) | 3129 ( 29.0 ) |
|  | hypotension* | 5662 ( 74.7 ) | 1922 ( 25.3 ) | 1441 ( 64.0 ) | 809 ( 36.0 ) |
|  | diabetes with complication** | 998 ( 76.3 ) | 310 ( 23.7 ) | 167 ( 69.3 ) | 74 ( 30.7 ) |
|  | Alzheimer’s or dementia* | 13226 ( 75.7 ) | 4250 ( 24.3 ) | 10845 ( 69.2 ) | 4830 ( 30.8 ) |
|  | depression** | 5321 ( 80.4 ) | 1294 ( 19.6 ) | 1786 ( 69.4 ) | 788 ( 30.6 ) |
|  | delirium* | 4763 ( 74.5 ) | 1632 ( 25.5 ) | 2040 ( 67.5 ) | 981 ( 32.5 ) |

* significance level ≤0.01

† Comorbidities are identified by the presence of ICD-10 diagnosis codes from the hip fracture care spell, or any admissions in the year prior to the hip fracture care spell.

‡ I – normal healthy individual; II – mild systemic disease that does not limit activity; III – severe systemic disease that limits activity but is not incapacitating; IV-incapacitating systemic disease which is constantly life-threatening; V-moribund -not expected to survive 24 hours with or without surgery.

§ low (less than first quartile), medium (second and third quartile), or high (fourth quartile) volume at admission based on the average annual number of surgeries at the admitting hospital.

ǁ Rehabilitation unit/acute hospital/already in hospital/this hospital site/other hospital site of this trust/other hospital trust

¶ Q1 and Q3: the first and third quarters respectively

** Age, deprivation, ethnicity, presence of chronic obstructive pulmonary diseases, ischemic heart disease, cardiac dysrhythmias,diabetes and depression are not different between early and late mobilised patients among patients who were admitted to hospital from nursing care.

†† significance level ≤0.05

# Supplementary File 3

Figure S3-1: Discharge by timing of mobilisation among patients surgically treated for non-pathological first hip fracture from the additive model including exposure by dementia, delirium, prefracture ambulation, and prefracture residence


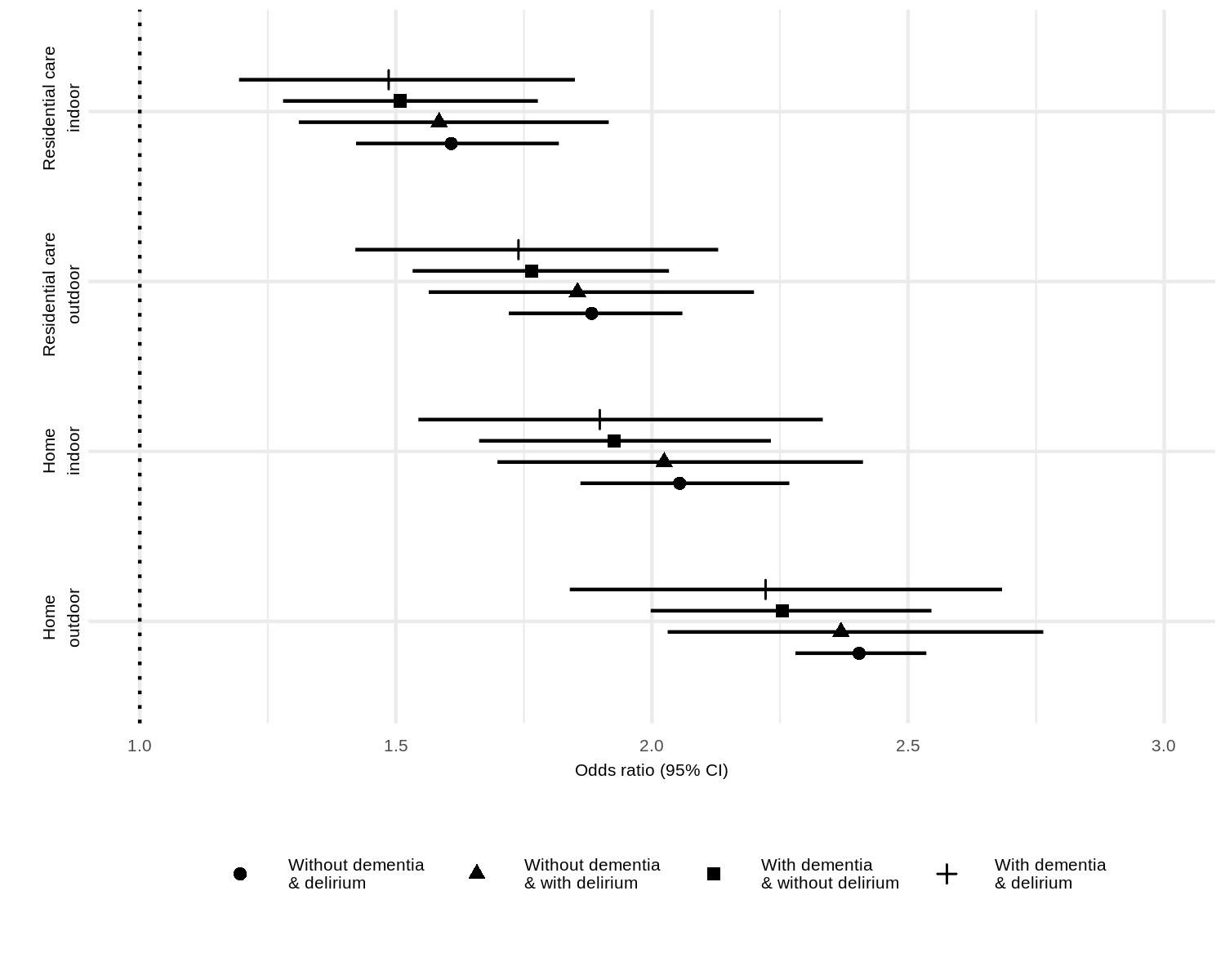


| **Prefracture residence** | **Prefracture ambulation** | **Dementia** | **Delirium** | **Mobilisation** | **No. of**  **patients** | **No of**  **deaths*** | **No. of live discharges**  **†** | **Unadjusted OR**  **of CIF (95% CI)** | **Adjusted OR of CIF (95% CI)**** |
| --- | --- | --- | --- | --- | --- | --- | --- | --- | --- |
| Residential Care | Indoor |  |  | Delayed | 571 | 73 | 322 | 1 | 1 |
| Residential Care | Indoor |  | With | Early | 1111 | 68 | 742 | 1.53  (1.28-1.84) | 1.49  (1.19-1.85) |
| Residential Care | Indoor | With |  | Delayed | 2166 | 199 | 1411 | 1 | 1 |
| Residential Care | Indoor |  | Without | Early | 4441 | 196 | 3273 | 1.41  (1.23-1.61) | 1.51  (1.28-1.78) |
| Residential Care | Indoor |  |  | Delayed | 0 | 0 | 0 | 1 | 1 |
| Residential Care | Indoor |  | With | Early | 0 | 0 | 0 | 2.05  (1.75-2.4) | 1.58  (1.31-1.92) |
| Residential Care | Indoor | Without |  | Delayed | 747 | 88 | 456 | 1 | 1 |
| Residential Care | Indoor |  | Without | Early | 1639 | 98 | 1108 | 1.89  (1.71-2.09) | 1.61  (1.42-1.82) |
| Residential Care | Outdoor |  |  | Delayed | 386 | 49 | 214 | 1 | 1 |
| Residential Care | Outdoor |  | With | Early | 884 | 56 | 587 | 1.81  (1.53-2.14) | 1.74  (1.42-2.13) |
| Residential Care | Outdoor | With |  | Delayed | 1592 | 118 | 1030 | 1 | 1 |
| Residential Care | Outdoor |  | Without | Early | 4192 | 148 | 3118 | 1.67  (1.49-1.87) | 1.77  (1.53-2.03) |
| Residential Care | Outdoor |  |  | Delayed | 0 | 0 | 0 | 1 | 1 |
| Residential Care | Outdoor |  | With | Early | 0 | 0 | 0 | 2.42  (2.1-2.79) | 1.85  (1.56-2.2) |
| Residential Care | Outdoor | Without |  | Delayed | 634 | 50 | 379 | 1 | 1 |
| Residential Care | Outdoor |  | Without | Early | 2053 | 96 | 1376 | 2.23  (2.07-2.4) | 1.88  (1.72-2.06) |
| Home | Indoor | With |  | Delayed | 594 | 81 | 117 | 1 | 1 |
| Home | Indoor | With | With | Early | 1228 | 96 | 378 | 1.98  (1.67-2.35) | 1.9  (1.54-2.33) |
| Home | Indoor | With |  | Delayed | 1052 | 145 | 257 | 1 | 1 |
| Home | Indoor | With | Without | Early | 2596 | 127 | 836 | 1.82  (1.62-2.05) | 1.93  (1.66-2.23) |
|  | Indoor | Without |  | Delayed | 0 | 0 | 0 | 1 | 1 |
|  | Indoor | Without | With | Early | 0 | 0 | 0 | 2.64  (2.28-3.06) | 2.02  (1.7-2.41) |
| Home | Indoor | Without |  | Delayed | 2684 | 339 | 673 | 1 | 1 |
| Home e | Indoor | Without | Without | Early | 7508 | 343 | 2724 | 2.44  (2.25-2.64) | 2.05  (1.86-2.27) |
| Home | Outdoor |  |  | Delayed | 1015 | 103 | 240 | 1 | 1 |
| Home | Outdoor |  | With | Early | 3484 | 182 | 1279 | 2.34  (2-2.73) | 2.22  (1.84-2.68) |
| Home | Outdoor | With |  | Delayed | 1512 | 141 | 424 | 1 | 1 |
| Home | Outdoor |  | Without | Early | 5723 | 222 | 2267 | 2.15  (1.95-2.37) | 2.26  (2-2.55) |
| Home | Outdoor |  |  | Delayed | 0 | 0 | 0 | 1 | 1 |
| Home | Outdoor |  | With | Early | 0 | 0 | 0 | 3.12  (2.74-3.56) | 2.37  (2.03-2.76) |
| Home | Outdoor | Without |  | Delayed | 10105 | 639 | 4221 | 1 | 1 |
| Home | Outdoor |  | Without | Early | 53136 | 907 | 31394 | 2.88  (2.76-3) | 2.4  (2.28-2.54) |

Figure S3-2: Discharge by timing of mobilisation among patients surgically treated for non-pathological first hip fracture from the additive model including exposure by dementia, prefracture ambulation, and prefracture residence


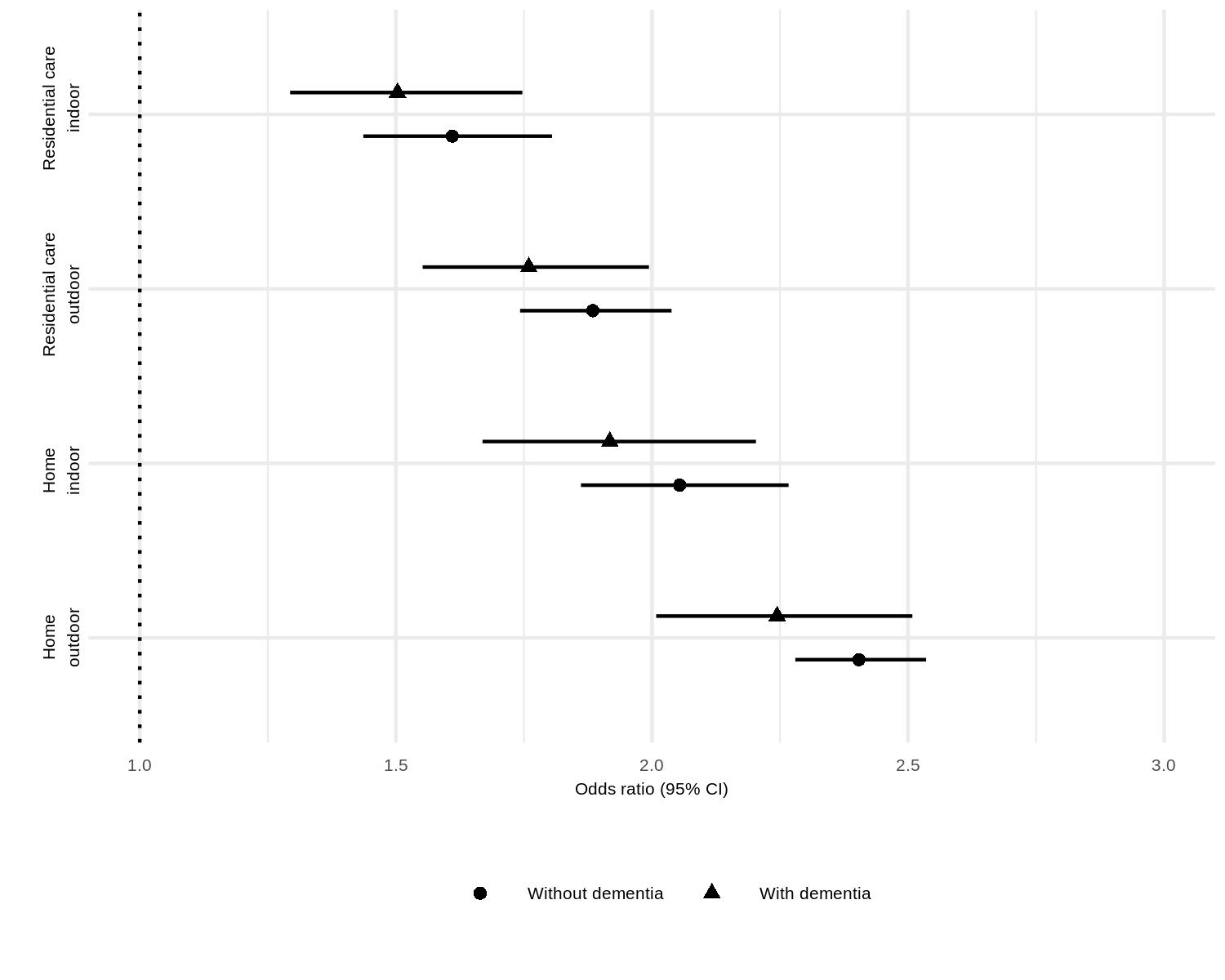


| **Prefracture residence** | **Prefracture ambulation** | **Dementia** | **Mobilisation** | **No. of**  **patients** | **No of**  **deaths*** | **No. of live discharges**  **†** | **Unadjusted OR**  **of CIF (95% CI)** | **Adjusted OR of CIF (95% CI)**** |
| --- | --- | --- | --- | --- | --- | --- | --- | --- |
|  |  | With | Delayed | 2737 | 272 | 1733 | 1 | 1 |
|  |  |  | Early | 5552 | 264 | 4015 | 1.43  (1.27 - 1.62) | 1.5  (1.29 - 1.75) |
|  | Indoor |  | Delayed | 747 | 88 | 456 | 1 | 1 |
| Residential Care |  | Without | Early | 1639 | 98 | 1108 | 1.88  (1.71 - 2.06) | 1.61  (1.44 - 1.8) |
|  |  | With | Delayed | 1978 | 167 | 1244 | 1 | 1 |
|  |  |  | Early | 5076 | 204 | 3705 | 1.69  (1.53 - 1.87) | 1.76  (1.55 - 1.99) |
|  | Outdoor |  | Delayed | 634 | 50 | 379 | 1 | 1 |
|  |  | Without | Early | 2053 | 96 | 1376 | 2.21  (2.08 - 2.36) | 1.88  (1.74 - 2.04) |
|  |  |  | Delayed | 1646 | 226 | 374 | 1 | 1 |
|  |  | With | Early | 3824 | 223 | 1214 | 1.86  (1.67 - 2.09) | 1.92  (1.67 - 2.2) |
|  | Indoor |  | Delayed | 2684 | 339 | 673 | 1 | 1 |
| Home |  | Without | Early | 7508 | 343 | 2724 | 2.44  (2.25 - 2.65) | 2.05  (1.86 - 2.27) |
|  |  |  | Delayed | 2527 | 244 | 664 | 1 | 1 |
|  |  | With | Early | 9207 | 404 | 3546 | 2.2  (2.01 - 2.41) | 2.24  (2.01 - 2.51) |
|  | Outdoor |  | Delayed | 10105 | 639 | 4221 | 1 | 1 |
|  |  | Without | Early | 53136 | 907 | 31394 | 2.88  (2.76 - 3.01) | 2.4  (2.28 - 2.54) |

# Supplementary File 4: Results from the analyses of the imputed data

## Sensitivity analysis results

Table S4-1: Imputation functions used in MICE process

| Variable | Method | Description | Scale type |
| --- | --- | --- | --- |
| AGE | Norm.nob | Linear Regression | continuous |
| SEXM, MOB_TIMING, FRACTYPE, RESIDENCE, PXTYPE, SX_YR, Sx_TIMING, DAYADM,  HRTFAIL, COPD, HYP, HYPO, CDYS, DIA, IHDA, IHDC, DEMTIA, DEPR, DELIRIUM | Logreg | Logistic regression | Factor, 2 levels |
| PREFRAFUN , ETHNIC, DEPRIVATION4, ASA | Polyreg | Polytomous regression | Factor, > 2 levels |

Table S4-2: Summary of combined results of cumulative incidence of live discharge by timing of mobilisation among patients surgically treated for non-pathological first hip fracture based on the sensitivity analysis using data imputation of missing information

| **Mobilisation timing** | **30-day CIF,**  **‡ (95% CI)** | **30-day Risk difference (95% CI)** | **Unadjusted OR**  **of CIF (95% CI)** * | **Adjusted OR of CIF (95% CI)** † |
| --- | --- | --- | --- | --- |
|  |  | **patients with Dementia** |  |  |
| Mobilised late | 532 (521-666) | -- | 1.00 | 1.00 |
| Mobilised early | 660 (653-578) | 60 (46-73) | 1.79 (1.7-1.89) | 1.86 (1.76-1.97) |
|  |  | **patients without Dementia** |  |  |
| Mobilised late | 569 (560-578) | -- | 1.00 | 1.00 |
| Mobilised early | 782 (778-544) | 140 (132-147) | 2.6 (2.51-2.7) | 2.27 (2.19-2.36) |
|  |  | **patients with Delirium** |  |  |
| Mobilised late | 420 (399-581) | -- | 1.00 | 1.00 |
| Mobilised early | 568 (555-575) | 80 (58-101) | 2.04 (1.82-2.29) | 2.05 (1.8-2.32) |
|  |  | **patients without Delirium** |  |  |
| Mobilised late | 570 (562-765) | -- | 1.00 | 1.00 |
| Mobilised early | 762 (758-440) | 114 (106-121) | 2.38 (2.31-2.46) | 2.14 (2.07-2.22) |
|  |  | **patients with Hypotension** |  |  |
| Mobilised late | 438 (417-658) | -- | 1.00 | 1.00 |
| Mobilised early | 644 (631-590) | 114 (93-134) | 2.41 (2.17-2.67) | 2.24 (1.99-2.52) |
|  | **patients without Hypotension** | | |  |
| Mobilised late | 567 (560-758) | -- | 1.00 | 1.00 |
| Mobilised early | 754 (751-459) | 111 (104-118) | 2.35 (2.27-2.43) | 2.12 (2.05-2.2) |
|  | **patients with indoor ambulation prefracture** | | |  |
| Mobilised late | 506 (495-652) | -- | 1.00 | 1.00 |
| Mobilised early | 644 (637-504) | 57 (45-69) | 1.88 (1.78-1.99) | 1.91 (1.8-2.03) |
|  | **patients with outdoor ambulation prefracture** | | |  |
| Mobilised late | 581 (572-781) | -- | 1.00 | 1.00 |
| Mobilised early | 777 (774-517) | 129 (121-138) | 2.44 (2.35-2.53) | 2.24 (2.15-2.32) |
|  | **patients admitted from residential care** | | |  |
| Mobilised late | 684 (673-809) | -- | 1.00 | 1.00 |
| Mobilised early | 803 (797-578) | 72 (59-85) | 1.79 (1.69-1.9) | 1.72 (1.61-1.83) |
|  |  | **patients admitted from home** |  |  |
| Mobilised late | 495 (486-734) | -- | 1.00 | 1.00 |
| Mobilised early | 731 (727-695) | 157 (149-165) | 2.8 (2.7-2.9) | 2.27 (2.19-2.36) |

Abbreviations: CIF = cumulative incidence function, CI = confidence interval, OR = odds ratio.

*The analysis includes those with known outcome and exposure. Data were imputed for patients whose exposure was missing, and/or potential confounders in the adjusted analyses.

† Adjusted for age, sex, ethnicity, fracture type, calendar period of admission, timing of surgery, comorbidity, prefracture residence, prefracture ambulation, procedure type, day of admission (not in the analysis by day of admission) and hospital volume. CIF regression at in-patient days 3, 4, 6, 8, 12, 16, 20, 24, and 30.

Table S4-3: Summary of combined results of cumulative incidence of live discharge by timing of mobilisation among patients surgically treated for non-pathological first hip fracture based on the sensitivity analysis based on the additive model with dementia, delirium, prefracture ambulation and prefracture residence using data imputation of missing information

| **Prefracture residence** | **Prefracture ambulation** | **Dementia** | **Delirium** | **Mobilisation** | **Unadjusted OR**  **of CIF (95% CI)** | **Adjusted OR of CIF (95% CI)*** |
| --- | --- | --- | --- | --- | --- | --- |
|  |  |  | With | Delayed | 1.00 | 1.00 |
|  |  |  | Without | Early | 1.7 (1.45-2) | 1.75 (1.47-2.07) |
|  |  |  | With | Delayed | 1.00 | 1.00 |
|  | Indoor | With | Without | Early | 1.52 (1.35-1.71) | 1.6 (1.42-1.81) |
|  |  |  | With | Delayed | 1.00 | 1.00 |
|  |  |  | Without | Early | 2.15 (1.86-2.48) | 1.96 (1.68-2.29) |
|  |  |  | With | Delayed | 1.00 | 1.00 |
| Residential care |  | Without | Without | Early | 1.92 (1.75-2.1) | 1.8 (1.63-1.98) |
|  |  |  | With | Delayed | 1.00 | 1.00 |
|  |  |  | Without | Early | 1.95 (1.68-2.28) | 1.88 (1.59-2.22) |
|  |  |  | With | Delayed | 1.00 | 1.00 |
|  | Outdoor | With | Without | Early | 1.75 (1.58-1.93) | 1.73 (1.55-1.92) |
|  |  |  | With | Delayed | 1.00 | 1.00 |
|  |  |  | Without | Early | 2.47 (2.15-2.83) | 2.11 (1.82-2.45) |
|  |  |  | With | Delayed | 1.00 | 1.00 |
|  |  | Without | Without | Early | 2.2 (2.06-2.36) | 1.94 (1.8-2.09) |
|  |  |  | With | Delayed | 1.00 | 1.00 |
|  |  |  | Without | Early | 2.13 (1.83-2.48) | 2.11 (1.8-2.49) |
|  |  |  | With | Delayed | 1.00 | 1.00 |
|  | Indoor | With | Without | Early | 1.91 (1.71-2.12) | 1.94 (1.73-2.18) |
|  |  |  | With | Delayed | 1.00 | 1.00 |
|  |  |  | Without | Early | 2.69 (2.36-3.07) | 2.37 (2.06-2.73) |
|  |  |  | With | Delayed | 1.00 | 1.00 |
| Home |  | Without | Without | Early | 2.4 (2.23-2.59) | 2.18 (2.02-2.35) |
|  |  |  | With | Delayed | 1.00 | 1.00 |
|  |  |  | Without | Early | 2.45 (2.12-2.82) | 2.28 (1.95-2.65) |
|  |  |  | With | Delayed | 1.00 | 1.00 |
|  | Outdoor | With | Without | Early | 2.19 (2-2.39) | 2.09 (1.9-2.3) |
|  |  |  | With | Delayed | 1.00 | 1.00 |
|  |  |  | Without | Early | 3.09 (2.73-3.49) | 2.55 (2.24-2.91) |
|  |  |  | With | Delayed | 1.00 | 1.00 |
|  |  | Without | Without | Early | 2.76 (2.65-2.87) | 2.34 (2.25-2.44) |

Abbreviations: CIF = cumulative incidence function, CI = confidence interval, OR = odds ratio.

* Adjusted for age, sex, ethnicity, fracture type, calendar period of admission, timing of surgery, comorbidity, procedure type, day of admission and hospital volume. CIF regression at in-patient days 3, 4, 6, 8, 12, 16, 20, 24, and 30. The results is based on the imputed data for 160,668 patients.

Table S4-4: Summary of combined results of cumulative incidence of live discharge by timing of mobilisation among patients surgically treated for non-pathological first hip fracture based on the sensitivity analysis based on the additive model with dementia, prefracture ambulation and prefracture residence using data imputation of missing information

| **Prefracture residence** | **Prefracture ambulation** | **Dementia** | **Mobilisation** | **Unadjusted OR**  **of CIF (95% CI)** | **Adjusted OR of CIF (95% CI)*** |
| --- | --- | --- | --- | --- | --- |
|  |  |  | Delayed | 1.00 | 1.00 |
|  | Indoor | With | Early | 1.56 (1.4-1.73) | 1.63 (1.46-1.83) |
|  |  |  | Delayed | 1.00 | 1.00 |
| Residential care |  | Without | Early | 1.91 (1.75-2.08) | 1.79 (1.63-1.96) |
|  |  |  | Delayed | 1.00 | 1.00 |
|  | Outdoor | With | Early | 1.78 (1.63-1.95) | 1.76 (1.6-1.94) |
|  |  |  | Delayed | 1.00 | 1.00 |
|  |  | Without | Early | 2.19 (2.05-2.33) | 1.92 (1.8-2.06) |
|  |  |  | Delayed | 1.00 | 1.00 |
|  | Indoor | With | Early | 1.97 (1.78-2.17) | 1.99 (1.79-2.21) |
|  |  |  | Delayed | 1.00 | 1.00 |
| Home |  | Without | Early | 2.41 (2.24-2.59) | 2.18 (2.02-2.35) |
|  |  |  | Delayed | 1.00 | 1.00 |
|  | Outdoor | With | Early | 2.25 (2.08-2.45) | 2.14 (1.96-2.34) |
|  |  |  | Delayed | 1.00 | 1.00 |
|  |  | Without | Early | 2.76 (2.65-2.88) | 2.35 (2.25-2.45) |

Abbreviations: CIF = cumulative incidence function, CI = confidence interval, OR = odds ratio.

* Adjusted for age, sex, ethnicity, fracture type, calendar period of admission, timing of surgery, comorbidity, procedure type, day of admission and hospital volume. CIF regression at in-patient days 3, 4, 6, 8, 12, 16, 20, 24, and 30. The results is based on the imputed data for 160,668 patients.

Table S4-5: Summary of combined results of cumulative incidence of live discharge by timing of mobilisation among patients surgically treated for non-pathological first hip fracture based on the sensitivity analysis based on the additive model with prefracture ambulation and prefracture residence using data imputation of missing information

| **Prefracture residence** | **Prefracture ambulation** | **Mobilisation** | **Unadjusted OR**  **of CIF (95% CI)** | **Adjusted OR of CIF (95% CI)*** |
| --- | --- | --- | --- | --- |
|  |  | Delayed | 1.00 | 1.00 |
|  | Indoor | Early | 1.60 (1.47 - 1.75) | 1.66 (1.51 - 1.82) |
| Residential |  | Delayed | 1.00 | 1.00 |
| care | Outdoor | Early | 1.91 (1.79 - 2.03) | 1.80 (1.68 - 1.93) |
|  |  | Delayed | 1.00 | 1.00 |
| Home | Indoor | Early | 2.29 (2.13 - 2.47) | 2.13 (1.97 - 2.30) |
|  |  | Delayed | 1.00 | 1.00 |
|  | Outdoor | Early | 2.73 (2.63 - 2.84) | 2.31 (2.22 - 2.41) |

Abbreviations: CIF = cumulative incidence function, CI = confidence interval, OR = odds ratio.

* Adjusted for age, sex, ethnicity, fracture type, calendar period of admission, timing of surgery, comorbidity, procedure type, day of admission and hospital volume. CIF regression at in-patient days 3, 4, 6, 8, 12, 16, 20, 24, and 30. The results is based on the imputed data for 160,668 patients.
